# Biochemical Changes during the Manufacture of Galician Chorizo Sausage as Affected by the Addition of Autochthonous Starter Cultures

**DOI:** 10.3390/foods9121813

**Published:** 2020-12-07

**Authors:** Miriam Rodríguez-González, Sonia Fonseca, Juan A. Centeno, Javier Carballo

**Affiliations:** 1 Food Technology Area, Faculty of Sciences, University of Vigo, 32004 Ourense, Spain; mirrodriguez@uvigo.es (M.R.-G.); soniafonseca@uvigo.es (S.F.); jcenteno@uvigo.es (J.A.C.); 2 CITACA, Agri-Food Research and Transfer Cluster, Campus da Auga, University of Vigo, 32004 Ourense, Spain

**Keywords:** Galician chorizo, starter cultures, *Staphylococcus equorum*, *Staphylococcus saprophyticus*, *Lactobacillus sakei*, physicochemical characteristics, free amino acids, free fatty acids, biogenic amines

## Abstract

In this work, the effect of the use of two autochthonous starter cultures (*Lactobacillus sakei* LS131 + *Staphylococcus equorum* SA25 (EQU), or *L. sakei* LS131 + *Staphylococcus saprophyticus* SB12 (SAP)) on the physicochemical, microbiological, proteolytic and lipolytic changes taking place during the manufacture of Galician chorizo, a traditional Spanish sausage, was studied. Three different batches (control (CNT), EQU and SAP) were manufactured in triplicate and analysed during the manufacturing process (samples were taken and analysed at 0, 2, 5, 9, 14, 21 and 30 days of ripening) for proximate composition, pH, a_w_, colour parameters, nitrogen fractions, free amino acids, biogenic amines, fat parameters and free fatty acids. The use of either of these two starter cultures slightly but significantly reduced the pH values during the fermentation and increased the percentage of transformation to nitrosyl-heme pigments as well as the a* and b* values in the final products. The two starters significantly decreased the *Enterobacteriaceae* counts in the final product, but without this microbial group completely disappearing. Both starter cultures significantly increased the α-amino acidic nitrogen and the total basic volatile nitrogen fractions during manufacturing, also increasing the free amino acid content and reducing the total biogenic amine content by approximately 20%. The SAP starter enhanced the lipolytic processes, increasing the free fatty acid content. Due to their performances, these two starter cultures seem to be suitable for increasing the quality and safety of the Galician chorizo sausage.

## 1. Introduction

In 2018, 1,429,000 Mt of meat products were manufactured in Spain [1]. Cooked sausages were the major products (430,000 Mt), followed by dry-cured hams and forelegs (306,000 Mt), dry-fermented sausages (214,000 Mt), fresh and marinated products (200,000 Mt), cooked hams and forelegs (175,000 Mt) and pre-prepared dishes (104,000 Mt). Despite the strong domestic demand, 63,103 Mt of dry-fermented sausages and 49,138 Mt of dry-cured hams were exported to the international market in 2019. The dry-fermented sausages were the meat products whose production experienced a greater increase in the last five years (from 186,000 Mt in 2013 to 214,000 Mt in 2018), and also the product that registered the greatest increase in exports (from 40,218 Mt in 2013 to 63,103 Mt in 2019) [1]. Spanish dry-fermented sausages are very diverse in size, appearance and organoleptic and nutritional characteristics, reflecting the traditions as well as the diversity of preferences and climatic conditions in the different regions and areas [2].

Galician chorizo is the typical traditional sausage of Galicia (NW of Spain), the one that enjoys the greatest acceptance and the most widely produced and consumed in this region, being also abundantly consumed in other regions of Spain and foreign countries. Its physicochemical [3,4,5,6] and microbiological [7,8] characteristics have been reasonably studied and described. It is both artisanal and industrially produced. Industries have adapted the traditional manufacturing procedures, incorporating the modern technologies in distinct steps of the production process. However, although the manufacturing method is nowadays perfectly standardized, batches of Galician chorizo present in the markets are very diverse in organoleptic characteristics, reflecting, above all, the diversity of the quality of the raw materials used. This variability diminishes the acceptance by the consumers, limits its demand and hinders its expansion in national and international markets.

It is widely known that the organoleptic characteristics of dry-fermented sausages are the result of a series of biochemical changes that take place during maturation [9], promoted by the meat and fat’s autochthonous enzymes and by those from the microorganisms that grow during the fermentation and maturation processes [10,11]. Both enzymatic activities are modulated by the rest of the ingredients (salt, spices, additives, etc.) and by the environmental conditions (temperature and relative humidity) present during the production process. Therefore, the diversity of the organoleptic characteristics of the dry-fermented sausages mainly reflects the diversity of the microorganisms acting during the maturation process.

Taking into account all these considerations, the use of an appropriate starter culture seems to be the most feasible solution for the heterogeneity of the organoleptic characteristics of Galician chorizo. The use of starter cultures, generally composed by a lactic acid bacteria and a coagulase-negative staphylococci (CNS), is a common and effective practice in the manufacture of fermented sausages in order to improve the colour and flavour development, ensure safety and extend their shelf-life [12,13,14,15,16]. However, the use of a commercial non-autochthonous starter culture could have a negative impact on the sensory characteristics of the sausages, resulting in losses of the desirable particular organoleptic properties that characterize each type of sausage [17,18].

With the aim of developing a specific and appropriate starter culture for the Galician chorizo sausage, bacterial strains isolated from Galician traditional artisanal sausages were isolated and adequately characterized regarding their technological and safety properties [19,20,21]. The most suitable lactic acid bacteria and *Staphylococcaceae* strains were then selected, and mixed cultures of *Lactobacillus sakei* and diverse species of *Staphylococcus* were developed and monitored throughout the ripening of this sausage using molecular methods [22]. In this last work, it was possible to verify that these cultures are capable of being implanted in the sausages, dominating the spontaneous flora.

As one of the final steps of this whole work, the aim of the present study was to evaluate the effect of two of these autochthonous starter cultures, consisting of a combination of one strain of *Lactobacillus sakei* and a strain of *Staphylococcus equorum* or *Staphylococcus saprophyticus*, on the biochemical changes that take place during the sausage ripening and that were responsible for the organoleptic characteristics of the final product.

Apart from the novelty of developing and testing a specific starter culture for an economically relevant dry-fermented sausage, the main novelty of the present work is the use of *Staphylococcus equorum* and *Staphylococcus saprophyticus* as starter cultures. *Staphylococcus xylosus* and *S. carnosus* are the only *Staphylococcus* species assayed as starter culture until now [12,14,15,23,24,25,26]. Regarding the lactic acid bacteria, the use of *Lactobacillus sakei* as a starter culture is common [13,24,25,27,28], although *Lb. plantarum* [10,11,15,26], *Lactobacillus curvatus* [12,23,29] and *Pediococcus pentosaceus* [14,24] were in many other cases preferred.

## 2. Materials and Methods

### 2.1. Preparation of the Starter Cultures

In order to inoculate the Galician chorizo batches, one *Lactobacillus* strain (*L. sakei* LS131-CECT 8335-) and two *Staphylococcus* strains (*S. equorum* SA25-CECT 8337- and *S. saprophyticus* SB12-CECT 8336-) were used as starter cultures. These strains were previously isolated from artisanal Androlla and Botillo, two traditional sausages made in Galicia (NW of Spain), and appropriately identified in our laboratory. These strains were chosen from a large set of isolates after testing their suitable technological and safety properties [19,20,21]. In brief, the strain *Lactobacillus sakei* LS 131 has a mild acidifying activity. The strain *Staphylococcus equorum* SA25 is slightly lipolytic; it has a medium proteolytic activity on the sarcoplasmic proteins and a lack of hydrolytic activity on the myofibrillar proteins. The strain *Staphylococcus saprophyticus* SB12 has high lipolytic activity, high proteolytic activity on the myofibrillar proteins and a lack of activity on the sarcoplasmic proteins [21,30]. The *Lactobacillus* strain was subcultured on MRS broth (Oxoid Ltd., Basingstoke, UK) to a final volume of 500 mL with a concentration of 10^8^ CFU/mL, whereas the *Staphylococcus* strains were subcultured on BHI broth (Oxoid) to a final volume of 1000 mL with a concentration of 10^8^ CFU/mL. The cell concentration was assessed by interpolation into the correspondent growth curve of the values of absorbance measured at 600 nm. Next, cells were obtained (centrifugation at 4000× *g* for 5 min at 4 °C) and washed (with 0.85% NaCl sterile solution). The pellets of cells were finally resuspended in 40 mL of sterile distilled water before addition to the sausage batches.

### 2.2. Production of Sausages and Sampling

Following the traditional procedure, three different batches of Galician chorizo were manufactured in triplicate. Batches were designed according to the starter culture added: CNT batch (control not inoculated), EQU batch (inoculated with *L. sakei* CECT 8335 + *S. equorum* CECT 8337) and SAP batch (inoculated with *L. sakei* CECT 8335 + *S. saprophyticus* CECT 8336). *L. sakei* CECT 8335 was inoculated in the sausage mix in a concentration of 10^6^ CFU/g, while *S. equorum* CECT 8337 and *S. saprophyticus* CECT 8336 were added in an amount of 10^7^ CFU/g. The mix of sausages was formulated according to traditional procedures, including lean pork shoulder (80%), pork back fat (20%), sweet paprika (22 g/kg), spicy paprika (1 g/kg), garlic (4 g/kg), salt (15 g/kg) and water (40 mL/kg). Lean and back fat were firstly ground using a 10-mm diameter mincing plate and next mixed together with the other ingredients for 3 min under vacuum. The resulting mix was allowed to stand at 4 °C for 24 h and then stuffed into porcine gut of 36–38 mm in diameter. Sausages were initially fermented for 9 days (6 °C and 80% relative humidity) and then dry-ripened for another 21 days (12 °C and 75% RH). From each replicate of each batch, samples were taken for subsequent analysis at 0 (mix before stuffing), 2, 5, 9, 14, 21 and 30 days of ripening.

### 2.3. Microbial Analysis

Ten grams of sample were taken in triplicate from the mix before stuffing or from the inner of the sausages at the different sampling times. Samples were aseptically added to 40 mL of a sterile solution containing 0.1% peptone (Oxoid), 0.85% NaCl (Oxoid) and 1% Tween 80 (Panreac Química SLU, Barcelona, Spain), and then homogenized in a Masticator Classic blender (IUL Instruments, Barcelona, Spain) for 2 min at room temperature. Serial decimal dilutions in sterile peptone water (0.1% (*w/v*)) were prepared and poured or spread in the corresponding agar media. Total mesophilic aerobic bacteria were enumerated in standard plate count agar (SPCA) (Oxoid) after incubation at 30 °C for 72 h; staphylococci on mannitol salt agar (MSA) (Oxoid) incubated at 30 °C for 48 h; lactic acid bacteria (LAB) in pH 5.7 de Man, Rogosa, Sharpe (MRS) agar (Merck GmbH, Darmstadt, Germany), overlaid and incubated at 30 °C for 5 days; and *Enterobacteriaceae* in violet red bile glucose agar (VRBGA) (Oxoid), overlaid and incubated at 37 °C for 24 h. Counts were expressed as log CFU/g.

### 2.4. Determination of the Proximate Composition and Physico-Chemical Parameters

Moisture, fat, protein (Kjeldahl nitrogen × 6.25), ash and NaCl contents were assessed following the standards ISO 1442:1997 [31], ISO 1443:1973 [32], ISO 937:1978 [33], ISO 936:1998 [34] and ISO 1841-1:1996 [35], respectively. Water activity was measured using a Fast-lab device (GBX, Bourg-de-Péage, France). The pH values were measured with a pH meter GLP21 (Crison Instruments, S.A., Barcelona, Spain) after mixing 10 g of sample with 90 mL of distilled water. Titratable acidity, nitrosyl-heme pigments, total heme pigments and percentage of conversion to cured meat pigments were determined according to the procedures described by Zaika et al. [36]. Colour parameters were measured using a portable CR-400 colorimeter (Konica Minolta Sensing Inc., Osaka, Japan). The results were expressed in the CIELAB space [37] as lightness (L*), redness (a*) and yellowness (b*).

### 2.5. Determination of the Nitrogen Fractions, Free Amino Acids and Biogenic Amines

The total non-protein nitrogen (NPN), α-amino acidic nitrogen (NH_2_-N) and total basic volatile nitrogen (TBVN) were quantified following the methods of Johnson [38], Moore and Stein [39] and Pearson [40], respectively, after precipitation of the proteins with 0.6 N HClO_4_, according to the procedure described by De Ketelaere et al. [41].

The extraction of free amino acids was performed as described by Alonso et al. [42]. The identification and quantification were carried out by HPLC techniques, using the conditions described by Alonso et al. [42], with some minor modifications. The liquid chromatography equipment consisted of a SpectraSystem module (Thermo Finnigan, San José, CA, USA) equipped with a SCM1000 vacuum membrane degasser, a P4000 pump, an AS3000 automatic injector, a UV6000LP photodiode array detector and ChromQuest Chromatography Workstation software. Separation was made in a reversed phase C18 Ultrasphere 5-ODS, 4.6 mm × 250 mm column (Hichrom Ltd., Theale, Berkshire, UK). The temperature of the column was maintained at 50 ± 1 °C with a column heater (SpectraSystem 3000) and the wavelength of the detector was at 254 nm. The standards of the 22 amino acids were supplied by Sigma Chemical Co. (St Louis, MO, USA). All the samples and standards were injected at least in duplicate. Repeatability tests were carried out by injecting a sample and a standard six times consecutively in a day. Reproducibility tests were also performed by injecting the sample and the standard two times per day during three consecutive days under the same experimental conditions. No significant differences (*p* < 0.05) were observed among the results obtained in these tests. Data were expressed as mg/100 g of total solids (TS).

The extraction of the biogenic amines was performed following the method described by Eerola et al. [43]. The separation, identification and quantification were carried out by HPLC techniques also following the procedure described by Eerola et al. [43], using the HPLC equipment already described. The separation was carried out in a reversed phase C18 mod. Kromasil 100 column (25 cm, 4 mm ID) (Teknokroma S. Coop. C. Ltda., San Cugat del Vallés, Barcelona, Spain). The temperature of the column was set at 40 ± 1 °C and the wavelength of the detector at 254 nm. The chromatographic conditions used were those described by Lorenzo et al. [44]. A standard containing appropriate amounts of histamine, tyramine, tryptamine, 2-phenylethylamine, putrescine, cadaverine, spermidine, spermine and 1,7-diaminoheptane (acting this later as internal standard) was used for identification and quantification. All the samples and standards were injected at least in duplicate. Repeatability and reproducibility tests were also carried out as indicated for the free amino acid analysis and significant differences (*p* < 0.05) were also not found between the results obtained in these tests. The contents of each biogenic amine were expressed as mg/kg of TS. From the values of the individual biogenic amines, the biogenic amine index (BAI) and the total vasoactive biogenic amine content (TVBA) were calculated as indicated in the foot of the table that shows the amine content in the results section.

### 2.6. Determination of Fat Indexes and Free Fatty Acids

After the fat extraction following the procedure of Folch et al. [45], the fat acidity and the peroxide values were determined following the Spanish Official Standards UNE 50.011 and UNE 55.023, respectively [46]. The TBA (thiobarbituric acid) value was measured following the method of Tarladgis et al. [47], with some modifications. All parameters were measured at least in duplicate in each fat sample.

The separation of the free fatty acids from the total fat was carried out in NH_2_-aminopropyl mini-columns, according to the method described by Kaluzny et al. [48]. The fatty acid methylation was carried out following the procedure described by Shehata et al. [49], with some modifications. The separation, identification and quantification of the fatty acid methyl esters were performed by gas chromatography techniques in a Trace GC chromatograph (Thermo Finnigan, Austin, TX, USA) equipped with a split/splitless, an AI 3000 autoinjector and a flame ionisation detector. The samples were injected in split mode. The separation of the different fatty acids was carried out on an Innowax column (length 30 m, ID25 mm, film thickness 0.25 mm) (Agilent Technologies, Santa Clara, CA, USA). The temperature of the detector was set at 250 °C and that of the injector at 230 °C. The gases used were hydrogen (35 mL/min), air (350 mL/min) and helium (carrier gas) (30 mL/min). The chromatographic conditions and the procedures for identification and quantification of the individual free fatty acids were those described by Méndez-Cid et al. [50]. All samples and standards were injected at least in duplicate. Repeatability and reproducibility tests were also carried out as previously indicated for the free amino acid determination. The free fatty acid contents were expressed as mg/100 g of fat.

### 2.7. Statistical Analysis

In order to analyse significant differences among batches and ripening times in the parameters studied, an analysis of variance (ANOVA) was performed using the General Linear Model (GLM) procedure of the SPSS package, version 23.0 (IBM SPSS, Chicago, IL, USA). The analysis of each parameter and significance was given as *p* < 0.05, *p* < 0.01 and *p* < 0.001. To determine the correlations between variables, Pearson’s linear coefficient was used, employing the same SPSS package.

## 3. Results and Discussion

### 3.1. Effect on Physicochemical and Microbial Changes during the Manufacturing Process

**Figure 1:**
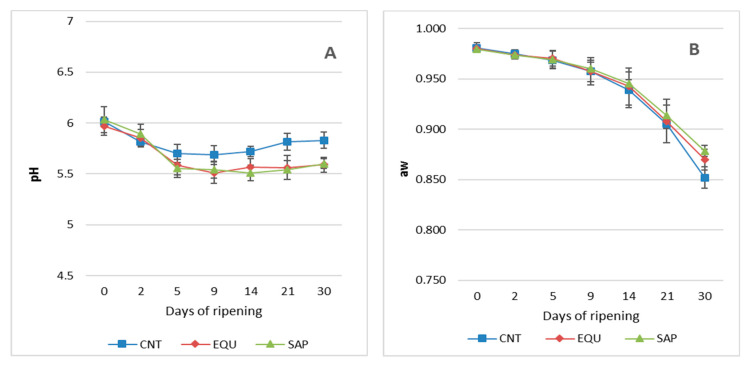
Evolution of the pH (**A**) and a_w_ (**B**) values along the manufacturing process of Galician sausage made without and with additives (plotted values are means ± standard deviations of three replicates in each sausage group). CNT: Non-inoculated control batches; EQU: Batches inoculated with *L. sakei* + *S. equorum*; SAP: Batches inoculated with *L. sakei* + *S. saprophyticus*.

Table 1 shows the values of the proximate composition of the sausage batches along the manufacturing process. The evolution of the a_w_ values is summarized in Figure 1B. The trends in moisture loss and the a_w_ decrease during the manufacture of the three sausage groups are very similar than those reported in the literature for other similar dry-fermented sausages [51,52,53] and are basically determined by the size of the sausages and by the environmental conditions (temperature and relative humidity) during the process. The protein, fat, ash and NaCl contents expressed as percentage of the total solids are within the wide range of values reported for similar sausage types and reflect the proportions of lean and fat used and the quantities of salt added in the mix preparation. None of these compositional parameters were significantly affected by the addition of starter cultures. The titratable acidity increased significantly from values of 0.15, 0.17 and 0.15 g of lactic acid/100 g of TS in the mix for the CNT, EQU and SAP batches, respectively, to values of 0.52, 0.69 and 0.71 g of lactic acid/100 g of TS (CNT, EQU and SAP batches, respectively) after 9 days of ripening, and then decreasing until reaching final values of 0.32, 0.59 and 0.67 g of lactic acid/100 g of TS, respectively. Significant differences (*p* < 0.001) were observed between the non-inoculated and inoculated batches. These values of titratable acidity are quite low, reflecting a moderate acidification during the manufacture of this sausage type. Information on the evolution of this parameter throughout the maturation of raw-cured sausages is not abundant in the literature, nor is there any discussion of the phenomena involved in such an evolution. The values in the present study are in accordance with that reported in previous works for similar sausages [52,53] and the trends in this parameter also agree with those indicated by Salgado et al. [53] in another variety of chorizo sausage. The decrease after day 14 of ripening is probably due to the consumption of organic acids by the microorganisms present, above all, moulds and yeasts. The increase in titratable acidity during the first 9 days of manufacturing and also the values reached were significantly (*p* < 0.001) higher in the batches manufactured using starter cultures than in the control, reflecting the acidifying capacity of the strain of *L. sakei* added.

The evolution of the pH values is shown in Figure 1A. Initial acidification plays an important role in the microbiological, biochemical and sensory characteristics of the fermented foods. In the case of the fermented sausages, the acidification until the pI of the muscle proteins causes denaturation of these proteins and determines the cohesiveness of the mass, the firmness and the sliceability of the final products [54]. The pH values reached, in addition, modulate the activity of the meat enzymes responsible for the ripening and flavour generation [55], and avoids the survival and growth of undesirable spoiling and pathogenic microorganisms.

In the present study, the decrease in pH values during the fermentation phase was very moderate (mean values from 6.02, 5.95 and 6.03 in the mix before stuffing to 5.69, 5.51 and 5.54 after 9 days of manufacturing, for the CNT, EQU and SAP batches, respectively). The pH decrease was significantly (*p* < 0.01) higher in batches of sausages inoculated with starter cultures, not observing significant differences between the two starter cultures used. From day 9 of manufacture, a slight and constant increase, more marked in the control sausages, was observed, reaching final average values of 5.81, 5.60 and 5.59 for the CNT, EQU and SAP batches, respectively. This pH increase in the last stages of the ripening process was already described by other authors in different sausages [15,52] and seems to be due to an increase of basic nitrogen compounds as a result of the proteolytic processes and also to the consumption of lactic acid by the microorganisms.

The pH decrease during fermentation is highly variable in the different sausages and depends on the quantity of fermentable sugars in the mix, the environmental temperature and the activity of the lactic acid bacteria present in the sausages. Therefore, the final pH values of the ripened sausages show a high variability, ranging along the values reported in the literature, from 4.15 [29] to 6.52 [56]. In agreement with some other previous observations [14], the use of starter cultures in the present study decreased the pH values during manufacturing by only a little. This contrasts with the great decrease observed in other studies where *Lactobacillus* strains with a greater acidifying capacity were used as starter cultures [29].

Colour is a very important sensory attribute in this type of food and colour deficiencies are likely to cause rejection even if sausages have a good taste and texture [57,58]. Table 2 shows the evolution of the colour parameters during the manufacture of the three batches of sausage. The percentage of conversion of pigments (from heme to nitrosyl-heme) had initial values of 35.16%, 39.63% and 37.56% for CNT, EQU and SAP sausages, respectively, with no significant (*p* > 0.05) differences between the three batches. As indicated in Table 2, the pigment transformation percentages showed a significant (*p* < 0.001) upward trend throughout the entire ripening process, reaching final values of 81,88% in the CNT batch and 82.02% and 86.26% in the EQU and SAP batches, respectively. The final value in the SAP batch was significantly (*p* < 0.05) higher than in the control batch. The values of percentage of transformation to nitrosyl-heme pigments in the present study are within the wide range of values reported in the literature [59] and reflect a high transformation of pigments. According to Zaika et al. [36], the percentage of pigment conversion is as high as the pH value is low, since the low pH values favour the formation of NO from nitrates that then reacts with myoglobin to form nitrosyl-myoglobin. Results in the present work seem to corroborate this appreciation, since in the present case the inoculated batches having lower pH values show higher conversion percentages. The nitrate reductase activity of the staphylococci strains added as starter cultures in the present work could also have some responsibility in the higher percentage of transformation in the inoculated batches.

Regarding the changes in the CIELAB colour coordinates (L*, a* and b*) throughout the drying–ripening process, the use of starter cultures did not have a significant effect on the luminosity (L*) of the sausages that decreased significantly (*p* < 0.05) during the whole drying–ripening process (values from 46–49 to 31–32), both in the control and in the inoculated batches. The decrease of this parameter during ripening seems to occur as a result of moisture loss [60,61,62], thus becoming a darker product. The evolution of the a* parameter (red coloration) is in line with the data described by Gómez et al. [58], with an increase up to 5 days of maturation, reaching a maximum value of 31.94 in the SAP batch, then decreasing to the end of the process with final values of 17.47, 20.70 and 20.25 in CNT, EQU and SAP, respectively. Significant differences were observed in this parameter both during ripening (*p* < 0.001) and due to the use of starter cultures (*p* < 0.05). The initial increase could be due to the formation of nitrosyl-myoglobin [63]. The initial redness is also influenced by the use of paprika in the mix formulation with a high colouring power, just as the oxidation of the carotenoids present in this ingredient contribute to the loss of coloration [58]. Finally, the parameter b * undergoes an evolution similar to that observed for redness. The initial increase in this parameter could be related to lipid oxidation processes. Again, we observed significant differences both during ripening (*p* < 0.001) and due to the use of starter cultures (*p* < 0.05).

Despite the fact that the correct implantation of these two starter cultures and their dominance over the indigenous microbiota was already observed and demonstrated using molecular methods [22], the main microbial groups were counted along the manufacture of the three sausage batches using classic culture-dependent procedures. Microbial counts are shown in Table 3. Counts of the microbial groups (5.48–6.57 log CFU/g for the total aerobic mesophilic bacteria, 4.82–6.42 log CFU/g for the total staphylococci, 4.12–5.47 log CFU/g for the lactic acid bacteria and 2.97–3.09 log CFU/g for the *Enterobacteriaceae* in the mix before stuffing) increased until the day 14 of ripening and then remained relatively constant or decreased in the case of the *Enterobacteriaceae* until the end of production. Counts and trends of the different microbial groups basically agree with previous data reported in the literature for similar sausages [12,64,65]. In each sampling time, counts of total aerobic bacteria, staphylococci, and lactic acid bacteria were always significantly (*p* < 0.001) higher in the inoculated batches than in the control batch, which confirmed the correct implantation of the starter cultures added. Higher counts of lactic acid bacteria and staphylococci in inoculated compared to control batches were also reported by Essid and Hassouna [15], using *Staphylococcus xylosus* and *Lactobacillus plantarum* as the starter cultures. Also, from day 14 of ripening, counts of the *Enterobacteriaceae* were significantly (*p* < 0.01) lower in the inoculated than in control sausages. This phenomenon seems to be due to the lower pH values reached in the inoculated sausages, taking into account the acid-sensitivity of the enterobacteria. In all three batches (CNT, EQU and SAP), the enterobacteria did not completely disappear at the end of maturation. In this sense, there is some discrepancies among the data reported in the literature. While some authors reported the total disappearance of the enterobacteria at the end of the ripening process in inoculated sausages having high pH values (5.63–5.76) [14], some others reported the survival of this microbial group in sausages reaching very low pH values (4.15) [15]. The different sources of contamination and the different nature and acid-resistance of the enterobacteria species present in the different sausage types could explain this discrepancy.

### 3.2. Effect on Proteolytic Changes during the Manufacturing Process

Hydrolysis of the meat proteins, both sarcoplasmic and myofibrillar, is considered one of the main degrading processes during the ripening of meat products playing a determinant role not only in the development of their final aroma and taste but also of the texture properties. Despite the fact that the electrophoretic methods, both the SDS-PAGE techniques [12,66,67] and miniaturized procedures [68], are the most exhaustive way of study the changes undergone by the proteins during the maturation processes, the quantification of the classical nitrogen fractions is a very satisfactory alternative.

Table 4 shows the values of these nitrogen fractions during the manufacture of the three batches of sausages. The non-protein nitrogen (126.23–133 mg/100 g of TS in the mix before stuffing) significantly increased (*p* < 0.001) during the manufacturing process, reaching final values of 167.45, 176.21 and 217.55 mg/100 g TS for the CNT, EQU and SAP sausages, respectively. At the end of the manufacture, values in the SAP batch were significantly (*p* < 0.001) higher than in the CNT and EQU batches. The increase in NPN is a common event that takes place in all the ripened sausage types during manufacturing, although in different rates and in unequal proportion during the different steps of the process, as indicated and discussed by Salgado et al. [53]. The low NPN contents in the mix before stuffing and the moderate increase observed in the present study (from 1.32 fold in CNT and EQU batches to 1.68 fold in the SAP batch) when compared to other dry-fermented sausages [53] indicate that proteolysis is only moderate in this type of sausage.

The α-aminoacidic nitrogen also increased significantly (*p* < 0.001) during the manufacturing, from 31.91, 44.48, and 42.71 to 109.73, 131.97 and 166.92 mg/100 g of TS for the CNT, EQU and SAP batches, respectively. Regarding the total basic volatile nitrogen, the increase was again significant (*p* < 0.001) from 13 mg/100 g of TS in the mix before stuffing to 83.05, 91.81 and 95.17 mg/100 g of TS for the CNT, EQU and SAP batches, respectively, at the end of the process. The values observed in the present study for these two nitrogen fractions are in the range of those reported in the literature for other similar sausage types [53,59].

Differences in NPN content among batches were not significant during the first 14 days of manufacture and only in the two last sampling times (21 and 30 days) the contents in the SAP batch were significantly (*p* < 0.001) higher than in the CNT and EQU batches. However, the contents in α-aminoacidic nitrogen were significantly (*p* < 0.001) higher in the inoculated than in the control batches in all the sampling times, and the same occurred for the total basic volatile nitrogen contents from day 2 of manufacture.

Although there is not a total consensus regarding the importance of the tissue and microbial enzymes in the protein degradation during sausage ripening, it seems evident that proteolysis takes place in two different phases. The initial protein degradation seems to be the responsibility of the muscle calpains and cathepsins, and at a later stage the bacterial enzymes further degrade the protein fragments and polypeptides initially formed [55]. The fact that in the present study the use of starter cultures had a greater effect on the α-aminoacidic and total basic volatile nitrogen fractions than on the non-protein nitrogen content seems to corroborate this hypothesis.

Release of free amino acids during sausage ripening is a very important event since some amino acids have a particular taste and some others are precursors of taste and odour compounds when degraded following several well-known biochemical pathways [61]. The contents of the free amino acids in the CNT, EQU and SAP sausage batches during manufacturing are shown in Table 5. Free amino acids in the mix before stuffing ranged from 428.12 in CNT batch to 444.53 mg/100 g TS in the SAP batch and no significant differences (*p* > 0.05) were observed among batches. A significant increase (*p* < 0.001) during manufacturing was observed in the three sausage groups reaching final values of 1381.66, 1450.02 and 1593.07 mg/100 g TS for the CNT, EQU and SAP batches, respectively. Significant differences (*p* < 0.05) among batches were observed in the final total free amino acid content. Therefore, from the initial and final values, an increase in the free amino acid content of 3.22, 3.33 and 3.58 times for the CNT, EQU and SAP batches can be observed, respectively. Similar increases were observed by other authors during the ripening of other sausage types [26,66]. Increases during ripening described in the literature are highly variable (around 1.2 times [12,27,69], around 1.5 times [65], around 2.5 times [23,61,70] or even more than 4 times [71]). There are, however, studies in which no increase [23] or a small reduction [12] was observed in control batches prepared in studies to determine the effect of the addition of starter cultures. As occurred in the present study, several authors reported that the addition of starter cultures always increased the release of free amino acids during the sausage ripening [14,15,23,26,71], which undoubtedly proves the participation of peptidases of microbial origin in the release of amino acids during the maturation of sausages.

The free amino acid profile in the mix before stuffing hardly varied among the batches. Arg was the most abundant FAA (73.21, 73.37 and 72.88 mg/100 g TS for the CNT, EQU and SAP batches, respectively), followed, in a decreasing order, by Tau, Ala, Pro, Lys and Leu, the sum of these six amino acids accounting for 61.09, 60.64 and 60.51% of the total FAA in the CNT, EQU and SAP batches, respectively. The free amino acid profile of the sausage mixes widely varies according to the information reported in the literature [12,23,61,65,66,72,73], reflecting the diversity of the operating microorganisms, environmental conditions and ingredients. However, in agreement with our observations, some other works [12,23,61,65,66,72,74] pointed out the abundance of Arg, Tau and Ala in the mix of the sausages. Indeed, Glu, which was reported as the main free amino acid in some studies [26,61,71], was the seventh or even the eighth free amino acid of quantitative importance in the mix before stuffing in the present study.

The individual FAA increased with a different intensity along the manufacturing process (from 2 times in the case of Tau, Arg or Ala to 6 times of Trp, or even 8–10 times of Cys). The most abundant FAA after 30 days of ripening was again Arg (151.09, 152.07 and 167.92 mg/100 g TS for the CNT, EQU and SAP batches, respectively), followed, in a decreasing order, by Ala, Tau, Glu, Pro and Leu. These six FAA accounted for 50.14, 49.65 and 50.13% of the total FAA in 30-day-old sausages in the CNT, EQU and SAP batches, respectively. The FAA profile observed in the mix basically remains in the ripened sausages, with the exception of Glu that increased its abundance and the Lys content that decreased. Again, the FAA profile of the ripened sausages is very variable in the literature [12,14,23,61,66,70,72]. However, in agreement with our results, the predominance of the Arg [61,74] and the abundance of Arg, Ala and Tau [12,23,65,66] was reported in other works.

The taste of some amino acids and their sensory thresholds were well stablished [75,76]. The amino acids Ala, Gly, Thr, Ser and Pro have a sweet taste. Leu, Val, Ile, Met and Phe are bitter. Glu, Asp and His have an acid taste and, in addition, Glu and Asp cause a pleasantly fresh sensation. Moreover, Asp, Tyr and Lys have been considered as responsible for an “aged” taste in the ripened meat products. On the basis of this knowledge, the FAA were grouped according their tastes (Table 5). In the ripened sausages (30 days) and when compared to the contents observed in mixes before stuffing, sweet FAA increased 3.33, 3.66 and 3.69 times in the CNT, EQU and SAP batches, respectively. Bitter FAA increased 3.70, 3.60 and 3.81 times in the CNT, EQU and SAP batches, respectively. Acid FAA experienced a more marked increase (4.79, 5.16 and 5.44 times, respectively), while “aged” FAA increased 3.66, 3.41 and 3.76 times in the CNT, EQU and SAP batches, respectively. In view of these data, it can be concluded that the use of *Lactobacillus sakei* and *Staphylococcus equorum* as starter cultures increased the sweet and acid tastes and decreased the bitter taste in the final product when compared with the non-inoculated control sausages. In the same way, the use of *Lactobacillus sakei* and *Staphylococcus saprophyticus* as starter cultures increased the four tastes when compared with the non-inoculated control sausages. Taking into account that, according to the information reported by Zhu [75], all these FAA are in the final sausages in concentrations higher than their respective sensory thresholds, the use of the starter cultures assayed in the present work could have some effect on the taste of the manufactured sausages.

Due to their importance for consumer health, derived from their physiological activities, and also due to their effects on the food quality, the biogenic amines are the products of the proteolysis in sausages that demanded more attention in the literature in the last two decades. Fermented sausages offer very favourable conditions for biogenic amine formation because of the high microbial activity during the fermentation process, the high presence of free amino acids (the biogenic amine precursors) as products of the proteolytic processes and the low acidic conditions that favour amino acid decarboxylation [77].

Table 6 shows the evolution of the main biogenic amines during the manufacture of the three Galician chorizo batches. In the present study, tyramine and spermine were the main biogenic amines in the mix, followed by tryptamine, 2-phenylethilamine, putrescine and cadaverine, with spermidine and histamine being the less abundant ones. The contents of most of these biogenic amines significantly (*p* < 0.001) increased during production but in an unequal way, with putrescine (that increased 4.79, 4.72 and 5.24 times in the CNT, EQU and SAP batches, respectively), histamine (4.46, 3.99 and 3.98 times) and cadaverine (4.07, 2.33 and 3.42 times) being the biogenic amines that underwent the greatest increases. Conversely, spermine (whose content did not experience a significant increase), spermidine (that increased 1.71, 1.76 and 1.63 times in the CNT, EQU and SAP batches, respectively) and 2-phenylethylamine (2.06, 1.81 and 1.97 times) were the amines that experienced the lowest increases. As a consequence of the individual amine increase, the total biogenic amine content increased 2.97, 2.48 and 2.53 times in the CNT, EQU and SAP batches, respectively, until reaching final total biogenic amine contents of 289.71, 241.47 and 235.32 mg/kg of TS in the CNT, EQU and SAP batches, respectively. These final content of total biogenic amines are in line with the contents described by other authors [13,68,74], although very much lower (61.71 mg/kg; [14]) and very much higher (1962.1 mg/kg; [72]) contents have been reported in the literature. In the final sausages, the main biogenic amine was putrescine, followed by tyramine, tryptamine and cadaverine. In general, tyramine, putrescine and cadaverine were reported as the main biogenic amines in meat products [24,78,79,80,81], with the concentration of cadaverine being the most variable [81]. Spermine and spermidine are the only biogenic amines present at significant levels in fresh meat [82]. According to the information reviewed by Suzzi and Gardini [79], several authors reported that strains of the genus *Lactococcus*, *Leuconostc* and *Lactobacillus* are able to produce tyramine, and therefore the generation of tyramine in sausages could be attributable to the decarboxylase activity of the lactic acid bacteria. Cadaverine and putrescine are associated with the activity of *Enterobacteriaceae* [79,83], and high quantities of these amines indicate poor hygienic practices and high microbial contamination of the raw materials [84]. In the present study, significant correlation coefficients (*r* = 0.692; *p* < 0.01) were observed between the counts in MRS agar and the tyramine contents, which seems to corroborate the responsibility of the lactic acid bacteria in the production of this amine. In the same way, a significant positive correlation (*r* = 0.727; *p* < 0.01) was observed between the counts in MRS agar and the tryptamine contents. The high significant correlations we observed between the counts in VRBGA and cadaverine (*r* = 0.635; *p* < 0.01) and putrescine (*r* + 0.560; *p* < 0.01) contents also suggests some implication of the *Enterobacteriaceae* in the generation of these two biogenic amines.

In the present study, the final content of total biogenic amines was significantly (*p* < 0.001) lower in the inoculated batches than in the control batch, and no significant differences (*p* > 0.05) were observed between the two inoculated batches. The use of starter cultures significantly reduced the total biogenic amine content, with the percentage of reduction being 16.65% in the EQU batch and 18.77% in the SAP batch. Reductions were unequal for the different biogenic amines, being cadaverine (45.03% and 36.26% of reduction for the EQU and SAP batches, respectively), tyramine (12.64% and 21.27%), 2-phenylethylamine (16.57% and 19.23%) and putrescine (12.47% and 17.51%) the amines that underwent the major reductions. In accordance with results of other previous studies [13], the spermine contents remained practically unaltered during the manufacturing process in the three sausage batches.

As indicated by Lorenzo et al. [81], several studies have demonstrated that the use of starter cultures reduce the biogenic amine formation during the sausage fermentation and ripening due to their inhibiting effect on the spoilage bacteria via acidification. However, some authors reported an increase in the biogenic amine content in the ripened sausages when some starter cultures were added [14,85]. This undesirable effect could be due to the increase of proteolysis and subsequent generation of free amino acids (precursors of the biogenic amines), although this possibility was questioned by the results of some studies [14,86]. Rather, it could be that the starter cultures favour the production of biogenic amines via a slight acidification that facilitates the decarboxylation reactions or by the direct production of biogenic amines by the strains integrating the starter cultures. In this sense, recent reports [87] indicated that the species *Staphylococcus xylosus*, largely used as starter culture in fermented sausages, is an effective producer of tyramine. In any case, some authors have indicated that a reduction in the formation of biogenic amines through acidification is only real for some biogenic amines, such as putrescine [85], and that a significant decrease in pH is necessary for this effect to occur. These authors indicated that the acidifying activity of the starter cultures did not reduce the tyramine production. However, our results regarding the tyramine reductions, as well as the results of some other authors concerning the reduction of this biogenic amine by using starter cultures [88,89], disagree with this statement. It seems, therefore, that more research is necessary to elucidate these discrepancies. It could be that other inhibitory mechanisms in addition to the pH reduction were involved in reducing the production of biogenic amines and that the strains that produce biogenic amines have a different sensitivity to these inhibitory mechanisms.

For additional information, the biogenic amine index (BAI) and the total vasoactive biogenic amines (TVBA) were calculated. Regarding the BAI, it was first developed and used by Mietz and Karmas [90], with the aim of assessing the freshness (bacterial quality) of tuna. The initial formula proposed by Mietz and Karmas does not take tyramine into account. In the present study, we used the formula for the BAI calculation proposed by Veciana-Nogués et al. [91], which does take into account this biogenic amine. Basically, a BAI quantifies the amines that come from microbial metabolism, and their evaluation is of great interest in foods in which any microbial growth is undesirable and indicates spoilage. In fermented foodstuffs (foods and beverages), there is a desirable and normal development of microorganisms during manufacturing. Therefore, these indices do not have an absolutely direct relationship with the microbiological quality of food. In the case of sausages, therefore, this index remains as an indicator of the degree of activity of the decarboxylating microorganisms in the product. The vasoactive amines (tyramine, histamine, tryptamine and 2-phenylethylamine) possess vasoactive and psychoactive properties and therefore indicate a food poisoning hazard. The use of starter cultures significantly reduced the BAI (19.66% in the EQU batch and 20.81% in the SAP batch) and the TVBA (12.12% and 16.23% in the EQU and SAP batches, respectively) in the final ripened sausages. This indicates that the use of these two starter cultures also improves the hygienic quality and safety of Galician chorizo sausage.

### 3.3. Effect on Lipolytic Changes during the Manufacturing Process

Lipolysis and fat oxidation are major sources of volatiles generated during the ripening of meat products. In order to investigate the effect of the addition of starter cultures on these chemical processes, we firstly analysed some parameters that indicate fat degradation. The results of these analyses are shown in Table 7. The acidity values that indicate the free fatty acid content increased significantly (*p* < 0.001) from 1.38, 1.32 and 1.47 mg KOH/g fat to 10.19, 10.05 and 13.39 mg KOH/g of fat in the CNT, EQU and SAP batches, respectively. At the end of the manufacturing process, the values were significantly (*p* < 0.001) higher in the SAP batch than in the CNT and EQU batches. This parameter therefore increased during the manufacture; specifically, 7.38, 7.61 and 9.10 times in the CNT, EQU and SAP batches, respectively. Increases of the acidity value reported in the literature in dry-fermented sausages are very variable. Similar increases than ours were observed by Salgado et al. [53] (10.42 times), but lower increases were reported by Lizaso et al. [64] (4.32 times), Franco et al. [52] (4 times) and Fernández-Fernández et al. [4] (1.7 times). The final values of fat acidity largely vary in the ripened sausages, as discussed by Franco et al. [52]. The final values in the present study are similar to those reported by Fernández-Fernández et al. [4] for the same sausage type, as well as to those observed by other authors in other ripened sausages [92,93]. These values indicate that this sausage undergoes during ripening a considerable lipolysis and that the use of the SAP starter enhances this process.

Peroxide values also increased significantly (*p* < 0.001), from 0.96, 1.10 and 1.18 to 6.02, 6.30 and 5.93 meq O_2_/kg fat in the CNT, EQU and SAP batches, respectively. At the end of ripening, no significant (*p* > 0.05) differences were observed among the batches. Usually, an increase in this parameter is noticed during sausage ripening [4,24,29,53]. However, a decrease was observed in other cases [94]; in some others, after an initial increase, a decline was reported in the last stages of ripening [29,52]. In the present study, the initial values of this parameter were low and, despite the fact that the peroxides increased during the manufacturing, the final values were lower than most of those reported in the literature for other similar sausages [24,29,52,53,64,92,95]. The reasons for the low values of this parameter in the present study could be the high quality of the fat used in the manufacture, coming from pigs slaughtered 48–72 h before manufacturing and adequately stored under refrigeration, and also the fact that the mincing, mixing and stuffing were carried out under vacuum, thus avoiding air (and therefore oxygen) incorporation during these processes.

The TBA value is a measure of the secondary oxidation processes and quantifies the malondialdehyde, one of the most representative compounds of those coming from the hydroperoxide decomposition. In the present study, the TBA value significantly (*p* < 0.001) increased from 0.16–0.22 to 0.54, 0.74 and 0.77 mg malondialdehyde/kg. The use of starter cultures significantly (*p* < 0.05) increased the value of this parameter in the final product and no differences (*p* > 0.05) were observed between the two inoculated batches. The evolution of the TBA value during the sausage ripening is very variable. As in the present study, Franco et al. [52] observed a progressive increase during the ripening process. However, in other cases after an initial increase, the malondialdehyde decreases in the final steps of the ripening [24,53,61,94]. Usually the concentration of malondialdehyde at the end of the manufacture of this type of sausages is around or under 1 mg/kg [25,52,53,61,94,95]. However higher values were frequently reported in the literature [24,92,95]. Domínguez Fernández and Zumalacárregui Rodríguez [96] reported values of 2.21 mg malondialdehyde/kg in “Chorizo” sausage after 35 days of ripening and indicated that this concentration is insufficient for the sensorial perception of rancidity.

Free fatty acid release during ripening of meat products is an important phenomenon for the sensory characteristics of the final products since most of the volatiles come from fatty acid degradation, mainly via oxidation processes. The evolution of the free fatty acids (FFA) during the manufacture of the batches of sausage is shown in Table 8. No significant differences (*p* > 0.05) were observed in the total FFA content among the batches in the mix. Total FFA contents in the mixes before stuffing (253.62, 247.11 and 263.64 mg/100 g of fat for the CNT, EQU and SAP batches, respectively) are in agreement with those reported in the literature for the mix of other sausages [12,26,61,70], although higher initial values were reported at the beginning of the manufacture of some other sausages [14,29,67]. Regarding the FFA profile of the mixes in the present work, the oleic acid (C18:1) was the main fatty acid, followed, in decreasing order of abundance, by linoleic (C18:2), palmitic (C16), and stearic (C18), these four FFA accounting for 87.40%, 87.07% and 87.32% of the total FFA of the mix in the CNT, EQU and SAP batches, respectively. The FFA profile in the mixes slightly differed among the batches. In the CNT batch, the fifth, sixth, seventh and eighth most important FFAs were the palmitoleic (C16:1), linolenic (C18:3), myristic (C14) and arachidonic (C20:4) fatty acids, while these same places were occupied by the linolenic C18:3), palmitoleic (C16:1), myristic (C14) and docosadienoic (C22:2) FA in the EQU batch, and by the linolenic (C18:3), palmitoleic (C16:1), arachidonic (C20:4) and myristic (C14) FA in the SAP batch. This FFA acid profile is quite constant in sausages manufactured from pig fat. Regarding the main fatty acids, profiles only differ in the FFA that occupy the second and third place. In some cases, palmitic (C16) is more abundant than linoleic (C18:2) [26,64], and in some other, as in the present work, linoleic (C18:2) dominates over palmitic (C16) [12,23,70]. In any case, very small differences between the palmitic (C16) and linoleic (C18:2) fatty acid concentrations have been consistently reported.

The total FFA content increased to final values of 1814.41, 1805.65 and 2295.46 mg/100 g of fat, for the CNT, EQU and SAP batches, respectively. In agreement with the observations made in the acidity values of the fat (Table 7), the final total FFA content was significantly (*p* < 0.001) higher in the SAP than in the EQU and CNT batches, and no significant (*p* > 0.05) differences were observed between the CNT and EQU batches. The increases in FFA content (7.15, 7.30 and 8.70 times for the CNT, EQU and SAP batches, respectively) are in agreement with the increases reported by other authors (6–8 times [12], 6–7 times [29] and 6 times [26]). However, lower [61,67,70] and higher [12] increases have been reported in other works. Usually, the use of starter cultures increases the FFA content during the ripening process. However, in some cases lower FFA contents were reported when commercial starter cultures were added [14].

In the present study, during the ripening process the different free fatty acids increased at a different rate (e.g., eicosatrienoic acid (C20:3n3) increased 10.88, 21.34 and 25.13 times in the CNT, EQU and SAP batches, respectively, while lauric acid (C12) only increased 3.14, 3.45, and 5.36 times in the CNT, EQU and SAP batches, respectively). The final values of total FFA content in the present work are in agreement with the values observed in other studies [12,67]. However, as occurred for the mixes, lower [26,61,70] and higher [14,29] values than ours have been reported.

The FFA profile in the ripened sausages hardly varied with respect to the profile in the mixes and in the different batches. The main FFA was again oleic acid (18:1), followed by linoleic (C18:2), palmitic (C16) and stearic (C18) acid, these four fatty acids accounting for 86.48, 83.56 and 87.53% of the total FFA in the CNT, EQU and SAP batches, respectively. The other FFA, in descending order of quantitative importance, were palmitoleic (C16:1), arachidonic (C20:4), docosadienoic (C22:2), linolenic (C18:3) and myristic (C14). The FFA profile in the ripened sausages basically agreed with that reported by other authors in other dry-fermented sausages [12,23,26,64,70,92] and hardly varies between the different authors and works when pork and pig fat are used in the sausage manufacture. This profile notably varies, however, when other fats such as tallow [25] or hump fat [28] are used. The FFA acid profile in the ripened sausages basically reflects the fatty acid composition of the fat of the raw materials and the nature of the lipases acting during the ripening process. Monounsaturated fatty acids always dominate in the FFA fraction of the sausages made from pork and pig fat since monounsaturated fatty acids dominate in the triacylglycerols that are the main fraction in the fat used for the manufacture. Some authors [97] reported that the majority of the FFA derives from the triacylglycerols. In this same line, Muriel et al. [98] also indicated that, in ripened meat products, the lipolytic processes affecting neutral lipids have a higher incidence in the FFA fraction than those affecting the polar lipids.

## 4. Conclusions

The use of autochthonous starter cultures integrated by *Lactobacillus sakei* LS131 + *Staphylococcus equorum* SA25 (starter EQU), or by *L. sakei* LS131 + *Staphylococcus saprophyticus* SB12 (starter SAP) in the manufacture of Galician chorizo slightly but significantly reduced the pH values during the fermentation and improved the colour by increasing the percentage of transformation to nitrosyl-heme pigments as well as the a* and b* values in the final products. The two starters also significantly decreased the *Enterobacteriaceae* counts in the final product, but without completely eliminating this microbial group.

Both starter cultures significantly increased the α-amino acidic nitrogen and the total basic volatile nitrogen fractions during manufacturing, also increasing the free amino acid content. Moreover, the two cultures reduced the total biogenic amine content by 20%, also reducing in the same proportion the total vasoactive biogenic amine content. The SAP starter enhanced the lipolytic processes, increasing the content in free fatty acids without modifying the FFA profile.

Due to their performances, these two starter cultures seem to be suitable for increasing the quality and safety of the Galician chorizo sausage.

## Figures and Tables

**Table 1:** Evolution of the proximate composition and titratable acidity along the manufacturing process of Galician chorizo made without and with starter cultures (means of three replicates in each sausage group).

Days of Ripening	0			2			5			9			14			21			30			SEM
	CNT	EQU	SAP	CNT	EQU	SAP	CNT	EQU	SAP	CNT	EQU	SAP	CNT	EQU	SAP	CNT	EQU	SAP	CNT	EQU	SAP	
TS ^#^	38.02 ^a,1^	38.08 ^a,1^	36.52 ^a,1^	42.72 ^b,1^	41.49 ^b,1^	40.81 ^b,1^	49.41 ^c,1^	48.10 ^c,1^	46.03 ^c,1^	53.14 ^d,1^	51.35 ^d,1^	51.14 ^d,1^	60.65 ^e,1^	59.10 ^e,1^	57.65 ^e,2^	68.04 ^f,1^	66.25 ^f,1^	67.42 ^f,1^	73.85 ^g,1^	71.80 ^g,1^	73.06 ^g,1^	2.14
Protein (N × 6.25) ^*^	49.22 ^a,1^	49.67 ^a,1^	48.34 ^a,1^	48.67 ^a,1^	49.08 ^a,1^	48.13 ^a,1^	48.47 ^a,1^	46.96 ^a,1^	48.63 ^a,1^	49.40 ^a,1^	48.39 ^a,1^	50.11 ^a,1^	48.92 ^a,1^	49.47 ^a,1^	50.42 ^a,1^	48.79 ^a,1^	50.59 ^a,1^	51.08 ^a,1^	50.75 ^a,1^	49.09 ^a,1^	51.22 ^a,1^	0.99
Fat ^*^	41.22 ^a,1^	41.97 ^a,1^	42.96 ^a,1^	41.88 ^a,1^	42.94 ^a,1^	42.42 ^a,1^	41.46 ^a,1^	43.12 ^a,1^	42.53 ^a,1^	41.65 ^a1^	42.13 ^a1^	42.87 ^a,1^	42.04 ^a,1^	43.25 ^a,1^	42.66 ^a,1^	42.92 ^a,1^	42.01 ^a,1^	42.13 ^a,1^	41.91 ^a,1^	42.30 ^a,1^	41.27 ^a,1^	0.36
Ash ^*^	6.13 ^a,1^	5.76 ^a,1^	6.09 ^a,1^	5.84 ^a,1^	6.01 ^a,1^	5.83 ^a,1^	5.58 ^a,1^	6.04 ^a,1^	5.87 ^a,1^	5.37 ^a,1^	6.82 ^b,2^	5.42 ^a,1^	6.15 ^a,1^	6.12 ^a,1^	5.91 ^a,1^	5.40 ^a,1^	6.41 ^a,1^	5.84 ^a,1^	5.54 ^a,1^	6.94 ^b,2^	5.85 ^a,1^	0.057
NaCl ^*^	3.24 ^a,1^	3.35 ^a,1^	3.27 ^a,1^	3.56 ^a,1^	3.64 ^a,1^	3.42 ^a,1^	3.64 ^a,1^	3.26 ^a,1^	3.74 ^a,1^	3.48 ^a,1^	3.59 ^a,1^	3.07 ^a,1^	3.59 ^a,1^	3.52 ^a,1^	3.37 ^a,1^	3.37 ^a,1^	3.52 ^a,1^	3.54 ^a,1^	3.48 ^a,1^	3.41 ^a,1^	3.50 ^a,1^	0.05
Titratable acidity ^§^	0.15 ^a,1^	0.17 ^a,1^	0.15 ^a,1^	0.32 ^b,1^	0.47 ^b,2^	0.49 ^b,2^	0.45 ^c,1^	0.67 ^c,2^	0.69 ^c,d,2^	0.52 ^d,1^	0.69 ^c,2^	0.71 ^d,2^	0.43 ^c,1^	0.72 ^d,2^	0.68 ^c,3^	0.45 ^c,1^	0.69 ^e,2^	0.67 ^c,2^	0.32 ^b,1^	0.59 ^e,2^	0.67 ^c,3^	0.02

^#^ Total solids expressed as g/100g; * Expresed as g/100g of TS.; ^§^ Expressed as g of lactic acid/100g of TS. CNT: Non-inoculated control batches; EQU: Batches inoculated with *L. sakei* + *S. equorum*; SAP: Batches inoculated with *L. sakei* + *S. saprophyticus*. ^a–g^ Means in the same row and sausage group (CNT, EQU or SAP) not followed by a common letter differ significantly (*p* < 0.05) (differences associated to the ripening time). ^1–3^ Means in the same row and ripening time not followed by a common number differ significantly (*p* < 0.05) (differences associated to the use of starter cultures). SEM: standard error of the mean.

**Table 2:** Evolution of the colour parameters along the manufacturing process of Galician chorizo made without and with starter cultures (means of three replicates in each sausage group).

Days of Ripening	0			2			5			9			14			21			30			SEM
	CNT	EQU	SAP	CNT	EQU	SAP	CNT	EQU	SAP	CNT	EQU	SAP	CNT	EQU	SAP	CNT	EQU	SAP	CNT	EQU	SAP	
Nitrosyl-heme pigments ^#^	23.89 ^a,1^	26.78 ^a,2^	25.29 ^a,3^	31.81 ^b,1^	37.21 ^b,2^	30.63 ^b,3^	41.04 ^c,1^	54.04 ^c,2^	54.54 ^c,2^	60.96 ^d,1^	69.73 ^d,2^	70.75 ^d,2^	71.48 ^e,1^	79.96 ^e,2^	91.86 ^e,3^	101.67 ^f,1^	104.98 ^f,2^	120.18 ^f,3^	111.67 ^g,1^	120.75 ^g,2^	128.16 ^g,3^	3.08
Total heme pigments ^#^	77.90 ^a,1^	70.70 ^a,2^	60.98 ^a,3^	83.05 ^b,1^	85.28 ^b,2^	69.42 ^b,3^	90.39 ^c,1^	107.95 ^c,2^	86.54 ^c,3^	115.62 ^d,1^	118.85 ^d,2^	104.47 ^d,3^	125.63 ^e,1^	128.53 ^e,2^	126.15 ^e,1^	138.54 ^f,1^	140.44 ^f,2^	159.74 ^f,3^	140.22 ^g,1^	146.66 ^g,2^	143.97 ^g,3^	2.65
P.C. ^§^	35.16 ^a,1^	39.63 ^a,2^	37.56 ^a,3^	38.46 ^b,1^	42.37 ^b,2^	43.97 ^b,3^	51.58 ^c,1^	51.15 ^c,1^	58.15 ^c,2^	62.82 ^d,1^	59.03 ^d,2^	58.68 ^d,2^	74.54 ^e,1^	66.57 ^e,2^	69.69 ^e,3^	75.34 ^e,1^	76.83 ^f,2^	73.27 ^f,3^	81.88 ^g,1^	82.02 ^g,1,2^	86.26 ^g,2^	1.45
L*	48.22 ^a,1^	49.86 ^a,2^	46.30 ^a,3^	50.68 ^b,1^	46.92 ^b,2^	52.05 ^b,1^	45.92 ^c,1^	45.60 ^c,1^	49.45 ^c,2^	44.54 ^d,1^	42.36 ^d,2^	43.39 ^d,1,2^	41.86 ^e,1^	43.16 ^e,2^	41.91 ^e,1^	36.26 ^f,1^	35.87 ^f,1^	35.68 ^f,1^	32.21 ^g,1^	31.39 ^g,1^	32.00 ^g,1^	0.62
a*	29.17 ^a,1^	29.00 ^a,1^	24.61 ^a,2^	27.67 ^b,1^	29.82 ^b,2^	31.01 ^b,3^	30.18 ^c,1^	30.16 ^c,1^	27.94 ^c,2^	30.18 ^c,1^	28.03 ^d,2^	25.18 ^d,3^	26.49 ^d,1^	29.91 ^e,2^	27.43 ^c,3^	21.44 ^e,1^	23.80 ^f,2^	24.03 ^f,2^	17.47 ^f,1^	20.70 ^g,2^	20.25 ^g,2^	0.38
b*	38.80 ^a,1,2^	39.05 ^a,2^	38.55 ^a,1^	41.17 ^b,1^	36.53 ^b,2^	44.35 ^b,3^	36.51 ^c,1^	40.10 ^c2^	40.55 ^c,2^	39.44 ^d,1^	35.51 ^d,2^	35.35 ^d,2^	28.60 ^e,1^	36.65 ^e,2^	30.72 ^e,3^	21.53 ^f,1^	27.54 ^f,2^	27.68 ^f,2^	19.91 ^g,1^	21.15 ^g,2^	20.48 ^g,3^	0.70

^#^ Expressed as ppm; ^§^ Percentage of transformation into nitrosylheme pigments. CNT: Non-inoculated control batches; EQU: Batches inoculated with *L. sakei* + *S. equorum*; SAP: Batches inoculated with *L. sakei* + *S. saprophyticus*. ^a–g^ Means in the same row and sausage group (CNT, EQU or SAP) not followed by a common letter differ significantly (*p* < 0.05) (differences associated to the ripening time). ^1–3^ Means in the same row and ripening time not followed by a common number differ significantly (*p* < 0.05) (differences associated to the use of starter cultures). SEM: standard error of the mean.

**Table 3:** Evolution of the plate counts (log CFU/g) of total aerobic mesophilic bacteria (SPCA), staphylococci (MSA), lactic acid bacteria (MRS) and *Enterobacteriaceae* (VRBGA) along the manufacturing process of Galician chorizo made without and with starter cultures (means of three replicates in each sausage group).

Days of Ripening	0			2			5			9			14			21			30			SEM
	CNT	EQU	SAP	CNT	EQU	SAP	CNT	EQU	SAP	CNT	EQU	SAP	CNT	EQU	SAP	CNT	EQU	SAP	CNT	EQU	SAP	
Total aerobic mesophilic bacteria	5.48 ^a,1^	6.54 ^a,2^	6.57 ^a,2^	5.93 ^b,1^	6.75 ^b,2^	7.24 ^b,3^	6.89 ^c,1^	7.93 ^c,2^	7.88 ^c,2^	8.37 ^d,1^	8.78 ^d,2^	8.99 ^d,3^	8.62 ^e,1^	8.97 ^e,2^	9.08 ^e,2^	8.78 ^f,1^	9.06 ^f,2^	9.19 ^f,2^	9.00 ^g,1^	9.12 ^g,2^	9.19 ^f,2^	0.16
Staphylococci	4.82 ^a,1^	6.26 ^a,2^	6.42 ^a,2^	4.71 ^b,1^	6.31 ^a,2^	6.75 ^b,3^	5.86 ^c,1^	6.90 ^b,2^	6.59 ^a,b,3^	6.40 ^d,1^	7.63 ^c,2^	7.53 ^c,2^	6.33 ^e,1^	8.05 ^d,2^	7.81 ^d,3^	6.24 ^f,1^	8.13 ^d,2^	7.95 ^d,2^	6.29 ^g,1^	8.08 ^d,2^	7.89 ^d,2^	0.15
Lactic acid bacteria	4.12 ^a,1^	5.47 ^a,2^	5.30 ^a,3^	4.96 ^b,1^	6.15 ^b,2^	5.80 ^b,3^	5.42 ^c,1^	6.36 ^c,2^	7.12 ^c,3^	7.35 ^d,1^	7.68 ^d,2^	7.86 ^d,3^	8.40 ^e,1^	8.98 ^e,2^	9.00 ^e,2^	8.37 ^f,1^	9.01 ^f,2^	9.15 ^f,3^	8.60 ^g,1^	9.14 ^g,2^	9.23 ^f,2^	0.14
*Enterobacteriaceae*	2.98 ^a,1^	2.97 ^a,1^	3.09 ^a,2^	3.56 ^b,1^	3.02 ^a,2^	3.28 ^b,3^	4.77 ^c,1^	4.91 ^b,2^	4.84 ^c,1,2^	5.42 ^d,1^	5.69 ^c,2^	5.36 ^d,1^	5.78 ^e,1^	5.47 ^d,2^	5.52 ^e,2^	5.00 ^f,1^	4.47 ^e,2^	4.62 ^f,3^	3.93 ^g,1^	3.59 ^f,2^	3.37 ^b,3^	0.15

CNT: Non-inoculated control batches; EQU: Batches inoculated with *L. sakei* + *S. equorum*; SAP: Batches inoculated with *L. sakei* + *S. saprophyticus*. ^a–g^ Means in the same row and sausage group (CNT, EQU or SAP) not followed by a common letter differ significantly (*p* < 0.05) (differences associated to the ripening time). ^1–3^ Means in the same row and ripening time not followed by a common number differ significantly (*p* < 0.05) (differences associated to the use of starter cultures). SEM: standard error of the mean.

**Table 4:** Evolution of the nitrogen fractions (expressed as mg N/100 g TS) along the manufacturing process of Galician chorizo made without and with starter cultures (means of three replicates in each sausage group).

Days of Ripening	0			2			5			9			14			21			30			SEM
	CNT	EQU	SAP	CNT	EQU	SAP	CNT	EQU	SAP	CNT	EQU	SAP	CNT	EQU	SAP	CNT	EQU	SAP	CNT	EQU	SAP	
Non-protein nitrogen (NPN)	126.23 ^a,1^	133.00 ^a,1^	129.45 ^a,1^	133.38 ^a,b,1^	150.57 ^b,1^	140.39 ^b,1^	148.81 ^c,1^	162.61 ^c,1^	139.41 ^b,1^	156.26 ^c,1^	163.30 ^c1^	154.46 ^c,1^	140.31 ^b,1^	166.05 ^c,1^	167.86 ^d,1^	156.41 ^c,1^	163.61 ^c,1^	193.02 ^e,2^	167.45 ^d,1^	176.21 ^d,1^	217.55 ^f,2^	7.95
α-Aminoacidic nitrogen (NH_2_-N)	31.91 ^a,1^	44.48 ^a,2^	42.71 ^a,3^	40.50 ^b,1^	50.17 ^b,2^	54.82 ^b,3^	46.46 ^c,1^	63.56 ^c,2^	66.07 ^c,3^	55.49 ^d,1^	76.64 ^d,2^	96.51 ^d,3^	59.74 ^e,1^	89.33 ^e,2^	117.83 ^e,3^	84.09 ^f,1^	106.47 ^f,2^	148.06 ^f,3^	109.73 ^g,1^	131.97 ^g,2^	166.92 ^g,3^	3.39
Total basic volatile nitrogen (TBVN)	13.33 ^a,1^	13.99 ^a,1^	13.40 ^a,1^	13.58 ^b,1^	17.75 ^b,2^	18.62 ^b,2^	23.50 ^c,1^	39.77 ^c,2^	32.90 ^c,3^	36.64 ^d,1^	58.27 ^d,2^	48.41 ^d,3^	53.79 ^e,1^	70.74 ^e,2^	66.08 ^e,3^	69.62 ^f,1^	79.74 ^f,2^	77.92 ^f,3^	83.05 ^g,1^	91.81 ^g,2^	95.17 ^g,3^	2.51

CNT: Non-inoculated control batches; EQU: Batches inoculated with *L. sakei* + *S. equorum*; SAP: Batches inoculated with *L. sakei* + *S. saprophyticus*. ^a–g^ Means in the same row and sausage group (CNT, EQU or SAP) not followed by a common letter differ significantly (*p* < 0.05) (differences associated to the ripening time). ^1–3^ Means in the same row and ripening time not followed by a common number differ significantly (*p* < 0.05) (differences associated to the use of starter cultures). SEM: standard error of the mean.

**Table 5:** Evolution of the free amino acids (mg/100g of TS) along the manufacturing process of Galician chorizo made without and with starter cultures (means of three replicates in each sausage group).

Days of Ripening	0			2			5			9			14			21			30			SEM
	CNT	EQU	SAP	CNT	EQU	SAP	CNT	EQU	SAP	CNT	EQU	SAP	CNT	EQU	SAP	CNT	EQU	SAP	CNT	EQU	SAP	
Asp	9.05 ^a,1^	9.34 ^a,1^	9.21 ^a,1^	13.12 ^b,1^	15.36 ^b,2^	17.72 ^b,3^	15.94 ^c,1^	18.88 ^c,2^	20.47 ^c,3^	16.43 ^d,1^	20.60 ^d,2^	22.29 ^d,3^	18.12 ^e,1^	21.83 ^e,2^	24.39 ^e,3^	21.49 ^f,1^	23.70 ^f,2^	28.41 ^f,3^	28.02 ^g,1^	29.27 ^g,3^	30.97 ^g,3^	0.55
Glu	19.03 ^a,1^	19.26 ^a,1^	19.80 ^a,1^	30.66 ^b,1^	37.34 ^b,2^	43.75 ^b,3^	53.86 ^c,1^	54.15 ^c,1^	68.55 ^c,2^	80.47 ^d,1^	91.52 ^d,2^	97.32 ^d,3^	85.92 ^e,1^	101.20 ^e,2^	104.38 ^e,3^	100.18 ^f,1^	113.17 ^f,2^	118.70 ^f,3^	108.12 ^g,1^	118.52 ^g,3^	126.47 ^g,3^	3.15
OH-Pro	4.87 ^a,1^	4.72 ^a,2^	4.74 ^a,2^	6.57 ^b,1^	7.63 ^b,2^	7.07 ^b,3^	9.20 ^c,1^	10.51 ^c,2^	14.17 ^c,3^	11.46 ^d,1^	14.97 ^d,2^	16.63 ^d,3^	13.99 ^e,1^	18.10 ^e,2^	19.27 ^e,3^	18.62 ^f,1^	20.90 ^f,2^	21.35 ^f,3^	23.03 ^g,1^	23.79 ^g,3^	25.76 ^g,3^	0.59
Asn	8.32 ^a,1^	9.37 ^a,2^	9.43 ^a,2^	11.62 ^b,1^	16.12 ^b,2^	18.99 ^b,3^	16.23 ^c,1^	21.26 ^c,2^	26.35 ^c,3^	18.48 ^d,1^	25.58 ^d,2^	28.92 ^d,3^	23.54 ^e,1^	31.34 ^e,2^	34.81 ^e,3^	29.93 ^f,1^	35.84 ^f,2^	39.03 ^f,3^	36.32 ^g,1^	41.73 ^g,3^	44.08 ^g,3^	0.96
Ser	11.12 ^a,1^	11.66 ^a,2^	11.60 ^a2^	14.18 ^b,1^	20.46 ^b,2^	26.00 ^b,3^	20.90 ^c,1^	35.47 ^c,2^	37.49 ^c,3^	33.70 ^d,1^	41.09 ^d,2^	45.43 ^d,3^	38.56 ^e,1^	49.23 ^e,2^	56.12 ^e,3^	47.28 ^f,1^	54.72 ^f,2^	56.97 ^f,3^	56.45 ^g,1^	63.05 ^g,3^	65.16 ^g,3^	1.54
Gln	7.89 ^a,1^	8.20 ^a,2^	8.34 ^a,2^	11.36 ^b,1^	13.11 ^b,2^	14.28 ^b,3^	15.20 ^c,1^	22.21 ^c,2^	24.15 ^c,3^	17.68 ^d,1^	27.79 ^d,2^	29.46 ^d,3^	20.71 ^e,1^	31.07 ^e,2^	33.90 ^e,3^	25.27 ^f,1^	36.02 ^f,2^	37.72 ^f,3^	29.38 ^g,1^	41.06 ^g,3^	44.29 ^g,3^	0.98
Gly	15.30 ^a,1^	14.47 ^a,2^	15.51 ^a,1^	23.62 ^b,1^	26.85 ^b,2^	30.10 ^b,3^	27.48 ^c,1^	45.27 ^c,2^	51.03 ^c,3^	39.28 ^d,1^	51.66 ^d,2^	55.73 ^d,3^	45.82 ^e,1^	57.30 ^e,2^	42.30 ^e,3^	54.11 ^f,1^	67.65 ^f,2^	72.26 ^f,3^	66.18 ^g,1^	73.76 ^g,3^	73.83 ^g,3^	1.73
His	8.15 ^a,1^	8.31 ^a,1^	8.22 ^a,1^	11.76 ^b,1^	14.25 ^b,2^	18.38 ^b,3^	17.33 ^c,1^	21.41 ^c,2^	28.64 ^c,3^	20.14 ^d,1^	27.85 ^d,2^	35.21 ^d,3^	23.56 ^e,1^	34.87 ^e,2^	37.68 ^e,3^	28.90 ^f,1^	38.13 ^f,2^	42.07 ^f,3^	37.45 ^g,1^	42.85 ^g,3^	45.39 ^g,3^	1.04
Tau	64.18 ^a,1^	65.87 ^a,2^	63.22 ^a3^	77.16 ^b,1^	79.10 ^b,2^	89.08 ^b,3^	82.03 ^c,1^	91.16 ^c,2^	100.51 ^c,3^	95.54 ^d,1^	106.81 ^d,2^	109.13 ^d,3^	99.60 ^e,1^	111.19 ^e,2^	113.40 ^e,3^	112.84 ^f,1^	116.16 ^f,2^	122.63 ^f,3^	121.80 ^g,1^	120.12 ^g,3^	146.44 ^g,3^	1.66
Arg	73.21 ^a,1^	73.37 ^a,1^	72.88 ^a,1^	93.27 ^b,1^	104.04^b,2^	117.43 ^b,3^	114.22 ^c,1^	125.07 ^c,2^	129.14 ^c,3^	120.07 ^d,1^	129.19 ^d,2^	135.98 ^d,3^	127.79 ^e,1^	131.61 ^e,2^	140.88 ^e,3^	138.56 ^f,1^	143.96 ^f,2^	148.39 ^f,3^	151.09 ^g,1^	152.07 ^g,1^	167.92 ^g,3^	2.15
Thr	9.25 ^a,1^	9.70 ^a,2^	9.87 ^a,2^	15.69 ^b,1^	19.93 ^b,2^	21.94 ^b,3^	16.84 ^c,1^	25.56 ^c,2^	30.44 ^c,3^	21.87 ^d,1^	32.50 ^d,2^	39.20 ^d,3^	23.23 ^e,1^	34.79 ^e,2^	40.91 ^e,3^	29.64 ^f,1^	39.23 ^f,2^	42.55 ^f,3^	34.07 ^g,1^	41.89 ^g,3^	45.04 ^g,3^	0.99
Ala	59.68 ^a,1^	60.08 ^a,1^	61.80 ^a,2^	68.46 ^b,1^	70.22 ^b,2^	74.98 ^b,3^	74.71 ^c,1^	95.72 ^c,2^	94.99 ^c,2^	86.20 ^d,1^	98.10 ^d,2^	102.84 ^d,3^	110.90 ^e,1^	115.89 ^e,2^	125.01 ^e,3^	122.45 ^f,1^	122.91 ^f,1^	134.34 ^f,3^	132.01 ^g,1^	135.77 ^g,3^	153.46 ^g,3^	2.42
Pro	21.92 ^a,1^	20.17 ^a,2^	24.27 ^a,3^	33.29 ^b,1^	41.31 ^b,2^	43.86 ^b,3^	48.31 ^c,1^	60.26 ^c,2^	65.79 ^c,3^	74.11 ^d,1^	90.25 ^d,2^	93.00 ^d,3^	82.60 ^e,1^	92.09 ^e,2^	95.53 ^e,3^	99.03 ^f,1^	102.52 ^f,2^	109.01 ^f,3^	102.54 ^g,1^	111.44 ^g,3^	117.57 ^g,3^	2.76
Tyr	9.02 ^a,1^	9.45 ^a,1^	9.40 ^a,1^	12.05 ^b,1^	14.13 ^b,2^	16.57 ^b,3^	16.14 ^c,1^	22.27 ^c,2^	26.77 ^c,3^	21.53 ^d,1^	29.08 ^d,2^	35.01 ^d,3^	25.83 ^e,1^	35.43 ^e,2^	37.41 ^e,3^	34.52 ^f,1^	37.96 ^f,2^	40.53 ^f,3^	47.23 ^g,1^	41.24 ^g,3^	52.24 ^g,3^	1.19
Val	19.13 ^a,1^	18.21 ^a,2^	18.88 ^a,12^	25.93 ^b,1^	31.88 ^b,2^	35.73 ^b,3^	37.84 ^c,1^	41.51 ^c,2^	44.16 ^c,3^	45.36 ^d,1^	46.76 ^d,2^	48.18 ^d,3^	46.35 ^e,1^	51.84 ^e,2^	54.12 ^e,3^	55.81 ^f,1^	57.74 ^f,2^	60.27 ^f,3^	69.83 ^g,1^	59.28 ^g,3^	66.88 ^g,3^	1.27
Met	9.32 ^a,1^	11.30 ^a,2^	12.49 ^a,3^	11.61 ^b,1^	19.99 ^b,2^	26.35 ^b,3^	14.66 ^c,1^	27.60 ^c,2^	35.46 ^c,3^	20.45 ^d,1^	31.02 ^d,2^	38.63 ^d,3^	26.89 ^e,1^	37.03 ^e,2^	42.89 ^e,3^	32.51 ^f,1^	41.63 ^f,2^	46.79 ^f,3^	41.55 ^g,1^	46.69 ^g,3^	55.43 ^g,3^	1.16
Cys	1.66 ^a,1^	1.74 ^a,1^	1.79 ^a,1^	4.35 ^b,1^	5.21 ^b,2^	6.19 ^b,3^	6.45 ^c,1^	10.80 ^c,2^	11.20 ^c,2^	8.07^d,1^	12.89 ^d,2^	15.69 ^d,3^	9.29 ^e,1^	13.57 ^e,2^	14.86 ^e,3^	11.59 ^f,1^	15.04 ^f,2^	16.18 ^f,3^	13.48 ^g,1^	15.75 ^g,3^	18.39 ^g,3^	0.47
Ile	13.27 ^a,1^	13.23 ^a,1^	11.88 ^a,2^	21.60 ^b,1^	22.06 ^b,1^	26.98 ^b,3^	25.95 ^c,1^	33.35 ^c,2^	36.44 ^c,3^	32.06 ^d,1^	36.23 ^d,2^	40.87 ^d,3^	37.09 ^e,1^	40.04 ^e,2^	45.45 ^e,3^	45.33 ^f,1^	45.55 ^f,1^	51.64 ^f,2^	55.82 ^g,1^	48.99 ^g,3^	53.96 ^g,3^	1.11
Leu	20.87 ^a,1^	20.56 ^a,1^	22.11 ^a,2^	39.38 ^b,1^	46.65 ^b,2^	46.86 ^b,2^	46.00 ^c,1^	58.73 ^c,2^	60.69 ^c,3^	53.53 ^d,1^	66.04 ^d,2^	65.50 ^d,2^	60.38 ^e,1^	70.60 ^e,2^	74.14 ^e,3^	66.66^f,1^	75.84 ^f,2^	84.66 ^f,3^	77.25 ^g,1^	82.14 ^g,3^	86.90 ^g,3^	1.68
Phe	15.80 ^a,1^	16.54 ^a,1^	18.18 ^a,2^	23.04 ^b,1^	25.19 ^b,2^	26.19 ^b,3^	27.06 ^c,1^	30.33 ^c,2^	34.32 ^c,3^	32.26 ^d,1^	36.21 ^d,2^	41.37 ^d,3^	33.68 ^e,1^	41.89 ^e,2^	45.80 ^e,3^	39.74 ^f,1^	45.93 ^f,2^	49.36 ^f,3^	46.05 ^g,1^	50.95 ^g,3^	55.64 ^g,3^	1.01
Trp	5.37 ^a,1^	5.69 ^a,12^	6.17 ^a,2^	6.77 ^b,1^	8.31 ^b,2^	12.36 ^b,3^	10.22 ^c,1^	12.28 ^c,2^	15.14 ^c,3^	22.18 ^d,1^	24.58 ^d,2^	26.76 ^d,3^	25.08 ^e,1^	26.24 ^e,2^	30.72 ^e,3^	29.45 ^f,1^	29.93 ^f,1^	34.65 ^f,2^	33.79 ^g,1^	34.87 ^g,3^	36.31 ^g,3^	0.97
Lys	21.71 ^a,1^	23.76 ^a,2^	24.74 ^a,3^	29.25 ^b,1^	32.87 ^b,2^	38.39 ^b,3^	37.40 ^c,1^	43.59 ^c,2^	47.86 ^c,3^	48.38 ^d,1^	52.30 ^d,2^	58.20 ^d,3^	53.66 ^e,1^	61.15 ^e,2^	69.24 ^e,3^	58.38 ^f,1^	66.22 ^f,2^	71.91 ^f,3^	70.24 ^g,1^	74.80 ^g,3^	80.93 ^g,3^	1.58
Total FAA	428.12 ^a,1^	435.00 ^a,1^	444.53 ^a,1^	584.74 ^b,1^	672.02 ^b,2^	759.21 ^b,3^	733.99 ^c,1^	907.39 ^c,2^	1003.77 ^c,3^	919.26 ^d,1^	1093.03 ^d,2^	1181.34 ^d,3^	1032.59 ^e,1^	1208.27 ^e,2^	1283.22 ^e,3^	1202.29 ^f,1^	1330.74 ^f,2^	1429.42 ^f,3^	1381.66 ^g,1^	1450.02 ^g,3^	1593.07 ^g,3^	30.01
∑ Sweet	117.26 ^a,1^	116.08 ^a,1^	123.05 ^a,2^	155.23 ^b,1^	178.77 ^b,2^	196.88 ^b,3^	188.24 ^c,1^	262.27 ^c,2^	279.75 ^c,3^	255.16 ^d,1^	313.61 ^d,2^	336.19^d,3^	301.11 ^e,1^	349.29 ^e,2^	359.87 ^e,3^	352.50 ^f,1^	387.03 ^f,2^	415.13 ^f,3^	391.24 ^g,1^	425.91 ^g,3^	455.06 ^g,3^	9.28
∑ Bitter	78.39 ^a,1^	79.84 ^a,1^	83.54 ^a,2^	121.57 ^b,1^	145.76 ^b,2^	162.11 ^b,3^	151.50 ^c,1^	191.53 ^c,2^	211.07^c,3^	183.66 ^d,1^	216.26 ^d,2^	234.54 ^d,3^	204.39 ^e,1^	241.39 ^e,2^	262.40 ^e,3^	240.05 ^f,1^	266.69 ^f,2^	292.72 ^f,3^	290.49 ^g,1^	288.04 ^g,3^	318.81 ^g,3^	6.07
∑ Acid	36.23 ^a,1^	36.91 ^a,1^	37.23 ^a,1^	55.55 ^b,1^	69.96 ^b,2^	79.85 ^b,3^	87.19 ^c,1^	94.44 ^c,2^	117.66 ^c,3^	117.04 ^d,1^	139.97 ^d,2^	154.82 ^d,3^	127.59 ^e,1^	157.90 ^e,2^	166.45 ^e,3^	150.58 ^f,1^	175.00 ^f,2^	189.18 ^f,3^	173.58 ^g,1^	190.64 ^g,3^	202.83 ^g,3^	4.66
∑ Aged	39.70 ^a,1^	42.55 ^a,2^	43.56 ^a,2^	54.43 ^b,1^	62.36 ^b,2^	72.68 ^b,3^	69.48 ^c,1^	84.74 ^c,2^	95.10 ^c,3^	86.34 ^d,1^	101.98 ^d,2^	115.50 ^d,3^	97.61 ^e,1^	118.41 ^e,2^	131.04 ^e,3^	114.39 ^f,1^	127.88 ^f,2^	140.85 ^f,3^	145.48 ^g,1^	145.31 ^g,1^	164.14 ^g,3^	3.19

∑ Sweet: sum of the sweet amino acids (Ala, Gly, Thr, Ser and Pro); ∑ Bitter: sum of the bitter amino acids (Leu, Val, Ile, Met and Phe); ∑ Acid: sum of the acid amino acids (Glu, Asp and His); ∑ Aged: sum of the amino acids responsible for the taste “aged” (Asp, Tyr and Lys). CNT: Non-inoculated control batches; EQU: Batches inoculated with *L. sakei* + *S. equorum*; SAP: Batches inoculated *with L. sakei* + *S. saprophyticus*. ^a–g^ Means in the same row and sausage group (CNT, EQU or SAP) not followed by a common letter differ significantly (*p* < 0.05) (differences associated to the ripening time). ^1–3^ Means in the same row and ripening time not followed by a common number differ significantly (*p* < 0.05) (differences associated to the use of starter cultures). SEM: standard error of the mean.

**Table 6:** Evolution of the biogenic amines (mg/kg of TS) along the manufacturing process of Galician chorizo made without and with starter cultures (means of three replicates in each sausage group).

Days of Ripening	0			2			5			9			14			21			30			SEM
	CNT	EQU	SAP	CNT	EQU	SAP	CNT	EQU	SAP	CNT	EQU	SAP	CNT	EQU	SAP	CNT	EQU	SAP	CNT	EQU	SAP	
Tryptamine	15.10 ^a,1^	14.91 ^a,1^	12.87 ^a,2^	19.84 ^b,1^	17.48 ^b,2^	16.55 ^b,3^	27.57 ^c,1^	21.22 ^c,2^	17.72 ^c,3^	37.02 ^d,1^	27.67 ^d,2^	25.37 ^d,3^	41.65 ^e,1^	30.23 ^e,2^	29.70 ^e,2^	40.15 ^f,1^	38.08 ^f,2^	33.43 ^f,3^	47.27 ^g,1^	41.69 ^g,3^	39.12 ^g,3^	10.63
2-Phenyl-ethylamine	12.40 ^a,1^	11.79 ^a,1^	10.44 ^a,2^	15.64 ^b,1^	14.08 ^b,2^	13.60 ^b,2^	18.91 ^c,1^	15.49 ^c,2^	14.04 ^c,3^	21.71 ^d,1^	15.46 ^c,2^	16.69 ^d,3^	24.94 ^e,1^	14.06 ^b,2^	20.81 ^e,3^	23.32 ^f,1^	20.85 ^d,2^	19.92 ^f,3^	25.58 ^g,1^	21.34 ^d,2^	20.66 ^g,3^	4.61
Putrescine	12.43 ^a,1^	11.04 ^a,2^	9.37 ^a,3^	19.99 ^b,1^	14.99 ^b,2^	10.96 ^b,3^	25.14 ^c,1^	23.95 ^c,2^	18.77 ^c,3^	32.80 ^d,1^	29.70 ^d,2^	23.18 ^d,3^	42.08 ^e,1^	38.01 ^e,2^	30.96 ^e,3^	50.92 ^f,1^	48.38 ^f,2^	40.38 ^f,3^	59.56 ^g,1^	52.13 ^g,2^	49.13 ^g,3^	15.51
Cadaverine	10.84 ^a,1^	10.38 ^a,1^	8.21 ^a,3^	16.22 ^b,1^	13.75 ^b,2^	9.73 ^b,3^	21.96 ^c,1^	16.57 ^c,2^	13.03 ^c,3^	27.49 ^d,1^	20.51 ^d,2^	17.09 ^d,3^	30.41 ^e,1^	22.41 ^e,2^	21.16 ^e,3^	39.92 ^f,1^	25.41 ^f,2^	23.34 ^f,3^	44.17 ^g,1^	24.28 ^g,2^	28.15 ^g,3^	9.47
Histamine	5.04 ^a,1^	5.27 ^a,1^	5.75 ^a,1^	7.09 ^b,1^	6.32 ^b,2^	7.44 ^b,1^	9.81 ^c,1^	7.44 ^c,2^	8.32 ^c,3^	11.96 ^d,1^	10.53 ^d,2^	11.88 ^d,1^	16.17 ^e,1^	12.99 ^e,2^	14.50 ^e,3^	20.11 ^f,1^	17.30 ^f,2^	15.91 ^f,3^	22.50 ^g,1^	21.04 ^g,2^	22.93 ^g,1^	5.86
Tyramine	21.90 ^a,1^	18.58 ^a,2^	19.59 ^a,3^	30.85 ^b,1^	20.66 ^b,2^	22.48 ^b,3^	33.75 ^c,1^	29.99 ^c,2^	28.55 ^c,3^	35.98 ^d,1^	31.78 ^d,2^	30.39 ^d,3^	38.83 ^e,1^	33.08 ^e,2^	32.47 ^e,2^	42.36 ^f,1^	43.37 ^f,1^	37.29 ^f,2^	56.46 ^g,1^	49.32 ^g,2^	44.45 ^g,3^	10.23
Spermidine	5.74 ^a,1^	5.44 ^a,1^	6.31 ^a,2^	6.09 ^a,1^	6.36 ^b,1^	6.56 ^a,1^	7.68 ^b,1^	7.47 ^c,1^	8.60 ^b,2^	7.56 ^b,1^	8.90 ^d,2^	8.59 ^b,2^	10.86 ^c,1^	10.10 ^e,12^	9.71 ^c,2^	8.93 ^d,1^	8.77 ^d,1^	9.49 ^c,1^	9.85 ^d,1,2^	9.59 ^e,1^	10.34 ^c,2^	3.42
Spermine	20.04 ^a,1^	19.65 ^a,1^	20.13 ^a,1^	23.58 ^b,1^	19.48 ^a,2^	21.37 ^a,2^	18.88 ^a1^	19.53 ^a,2^	17.94 ^b,3^	20.08 ^a,1^	22.00 ^b,2^	22.30 ^a,2^	20.26 ^a,1^	23.99 ^b,2^	23.18 ^a,2^	23.84 ^b,1^	25.83 ^c,2^	23.85 ^b,1^	24.32 ^b,1^	22.09 ^b,2^	20.53 ^a,2^	8.53
TBA	103.47 ^a,1^	97.07 ^a,2^	92.67 ^a,3^	139.30 ^b,1^	113.12 ^b,2^	108.70 ^b,3^	163.71 ^c,1^	141.66 ^c,2^	126.96 ^c,3^	194.60 ^d,1^	166.53 ^d,2^	155.49 ^d,3^	225.20 ^e,1^	184.88 ^e,2^	182.49 ^e,2^	249.55 ^f,1^	228.00 ^f,2^	203.60 ^f,3^	289.71 ^g,1^	241.47 ^g,2^	235.32 ^g,2^	65.45
BAI	50.20 ^a,1^	45.28 ^a,2^	42.92 ^a,3^	74.15 ^b,1^	55.72 ^b,2^	50.62 ^b,3^	90.66 ^c,1^	77.94 ^c,2^	68.67 ^c,3^	108.24 ^d,1^	92.52 ^d,2^	82.54 ^d,3^	127.49 ^e,1^	106.50 ^e,2^	99.10 ^e,3^	153.31 ^f,1^	134.46 ^f,2^	116.91 ^f,3^	182.69 ^g,1^	146.77 ^g,2^	144.67 ^g,2^	39.84
TVBA	54.44 ^a,1^	50.55 ^a,2^	48.66 ^a,3^	73.42 ^b,1^	58.54 ^b,2^	60.08 ^b,3^	90.04 ^c,1^	74.15 ^c,2^	68.63 ^c,3^	106.67 ^d,1^	85.43 ^d,2^	84.33 ^d,3^	121.59 ^e,1^	90.36 ^e,2^	97.48 ^e,3^	125.94 ^f,1^	119.60 ^f,2^	106.55 ^f,3^	151.80 ^g,1^	133.39 ^g,2^	127.16 ^g,2^	30.29

TBA: Total biogenic amines; BAI: Biogenic amine index (sum of putrescine + cadaverine + histamine+ tyramine); TVBA: sum of the vasoactive amines (tyramine + histamine + tryptamine + 2 phenylethylamine). CNT: Non-inoculated control batches; EQU: Batches inoculated with *L. sakei* + *S. equorum*; SAP: Batches inoculated with *L. sakei* + *S. saprophyticus*. ^a–g^ Means in the same row and sausage group (CNT, EQU or SAP) not followed by a common letter differ significantly (*p* < 0.05) (differences associated to the ripening time). ^1–3^ Means in the same row and ripening time not followed by a common number differ significantly (*p* < 0.05) (differences associated to the use of starter cultures). SEM: standard error of the mean.

**Table 7:** Evolution of the fat parameters along the manufacturing process of Galician chorizo made without and with starter cultures (means of three replicates in each sausage group).

Days of Ripening	0			2			5			9			14			21			30			SEM
	CNT	EQU	SAP	CNT	EQU	SAP	CNT	EQU	SAP	CNT	EQU	SAP	CNT	EQU	SAP	CNT	EQU	SAP	CNT	EQU	SAP	
Acidity index ^#^	1.38 ^a,1^	1.32 ^a,1^	1.47 ^a,2^	2.55 ^b,1^	3.12 ^b,2^	2.93 ^b,3^	3.61 ^c,1^	3.87 ^c,2^	3.65 ^c,1^	4.49 ^d,1^	4.65 ^d,2^	4.70 ^d,2^	6.09 ^e,1^	6.84 ^e,2^	6.11 ^e,1^	7.82 ^f,1^	8.94 ^f,2^	10.28 ^f,3^	10.19 ^g,1^	10.05 ^g,1^	13.39 ^g,2^	0.26
Peroxide value ^*^	0.96 ^a,1^	1.10 ^a,2^	1.18 ^a,3^	1.77 ^b,1^	2.13 ^b,2^	1.86 ^b,3^	3.18 ^c,1^	3.27 ^c,1^	2.43 ^c,2^	3.99 ^d,1^	4.17 ^d,2^	3.24 ^d,3^	4.05 ^e,1^	4.56 ^e,2^	4.17 ^e,1^	4.86 ^f,1^	5.07 ^f,2^	5.22 ^f,2^	6.02 ^g,1^	6.30 ^g,1^	5.93 ^g,1^	0.05
TBA value ^§^	0.20 ^a,1,2^	0.22 ^a,1^	0.16 ^a,2^	0.19 ^a,1^	0.17 ^b,1^	0.29 ^b,2^	0.18 ^a,1^	0.21 ^a,1^	0.28 ^b,2^	0.34 ^b,1^	0.21 ^a,2^	0.24 ^c,3^	0.46 ^c,1^	0.32 ^c,2^	0.34 ^d,2^	0.48 ^c,1^	0.37 ^d,2^	0.60 ^e,3^	0.54 ^d,1^	0.74 ^e,2^	0.77 ^f,2^	0.02

^#^ Expressed as mg KOH/g fat; * Expressed as meq de O_2_/kg fat; ^§^ Expressed as mg malondialdehyde/kg of sample; CNT: Non-inoculated control batches; EQU: Batches inoculated with *L. sakei* + *S. equorum*; SAP: Batches inoculated with *L. sakei* + *S. saprophyticus*. ^a–g^ Means in the same row and sausage group (CNT, EQU or SAP) not followed by a common letter differ significantly (*p* < 0.05) (differences associated to the ripening time). ^1–3^ Means in the same row and ripening time not followed by a common number differ significantly (*p* < 0.05) (differences associated to the use of starter cultures). SEM: standard error of the mean.

**Table 8:** Evolution of the free fatty acids (mg FFA/100g of fat) along the manufacturing process of Galician chorizo made without and with starter cultures (mean values of three batches in each sausage group).

Days of Ripening	0			2			5			9			14			21			30			SEM
	CNT	EQU	SAP	CNT	EQU	SAP	CNT	EQU	SAP	CNT	EQU	SAP	CNT	EQU	SAP	CNT	EQU	SAP	CNT	EQU	SAP	
C8	0.26 ^a,1,2^	0.28 ^a,1^	0.25 ^a,2^	0.26 ^a,1^	0.33 ^b,2^	0.38 ^b,3^	0.40 ^b,1^	0.52 ^c,2^	0.35 ^c,3^	0.53 ^c,1^	0.61 ^d,2^	0.46 ^d,3^	0.67 ^d,1^	0.74 ^e,2^	0.73 ^e,2^	1.11 ^e,1^	0.84 ^f,2^	0.91 ^f,3^	1.52 ^f,1^	1.36 ^g,2^	1.33 ^g,3^	0.03
C10	0.45 ^a,1^	0.25 ^a,2^	0.34 ^a,3^	0.46 ^a,1^	0.39 ^b,2^	0.45 ^b,1^	0.52 ^b,1^	0.61 ^c,2^	0.46 ^b,3^	0.64 ^c,1^	0.72 ^d,2^	0.60 ^c,3^	0.87 ^d,1^	1.05 ^e,2^	1.07 ^d,2^	1.21 ^e,1^	1.44 ^f,2^	1.92 ^e,3^	1.37 ^f,1^	1.93 ^g,2^	2.49 ^f,3^	0.05
C12	0.69 ^a,1^	0.61 ^a,2^	0.55 ^a,3^	0.74 ^b,1^	0.68 ^b,2^	0.86 ^b,3^	0.75 ^b,1^	0.84 ^c,2^	1.17 ^c,3^	0.91 ^c,1^	1.13 ^d,2^	1.29^d3^	1.02 ^d,1^	1.48 ^e,2^	1.54 ^e,3^	1.38 ^e,1^	1.51 ^f,2^	1.98 ^f,3^	2.17 ^f,1^	2.11 ^g,2^	2.95 ^g,3^	0.05
C14	4.59 ^a,1^	3.58 ^a,2^	3.22 ^a,3^	4.51 ^a,1^	5.55 ^b,2^	5.54 ^b,2^	5.11 ^b,1^	6.48 ^c,2^	6.95 ^c,3^	6.26 ^c,1^	8.24 ^d,2^	8.76^d3^	8.15 ^d,1^	12.03 ^e,2^	12.35 ^e,3^	12.09 ^e,1^	14.16 ^f,2^	18.22 ^f,3^	17.89 ^f,1^	18.69 ^g,2^	26.14 ^g,3^	0.49
C14:1	0.15 ^a,1^	0.18 ^a,2^	0.16 ^a,1,2^	0.21 ^b,1^	0.21 ^b,1^	0.15 ^a,2^	0.23 ^b,c,1^	0.24 ^c1^	0.20 ^b,2^	0.24 ^c,1^	0.38 ^d,2^	0.23^c1^	0.29 ^d,1^	0.48 ^e,2^	0.31 ^d,1^	0.37 ^e,1^	0.61 ^f,2^	0.41 ^e,3^	0.53 ^f,1^	0.71 ^g,2^	0.84 ^f,3^	0.02
C15	0.40 ^a,1^	0.39 ^a,1^	0.41 ^a,1^	0.42 ^a,1^	0.44 ^b,1^	0.48 ^b,2^	0.45 ^b,1^	0.72 ^c,2^	0.53 ^c,3^	0.52 ^c,1^	0.77 ^d,2^	0.65^d3^	0.58 ^d,1^	1.09 ^e,2^	1.05 ^e,2^	0.90 ^e,1^	1.34 ^f,2^	1.58 ^f,3^	1.19 ^f,1^	1.75 ^g,2^	1.98 ^g,3^	0.04
C15:1	0.20 ^a,1^	0.29 ^a,2^	0.21 ^a,1^	0.32 ^b,1^	0.31 ^a,1^	0.55 ^b,2^	0.62 ^c,1^	0.60 ^b,1^	0.56 ^b,2^	0.67 ^d,1^	0.88 ^c,2^	0.72^c,3^	0.95 ^e,1^	1.10 ^d,2^	1.04 ^d,3^	1.04 ^f,1^	1.31 ^e,2^	1.23 ^e,3^	1.32 ^g,1^	1.45 ^f,2^	1.29 ^f,3^	0.03
C16	53.63 ^a,1^	41.28 ^a,2^	50.01 ^a,3^	72.78 ^b,1^	93.76 ^b,2^	82.77 ^b,3^	91.58 ^c,1^	110.43 ^c,2^	123.56 ^c,3^	105.66 ^d,1^	134.64 ^d,2^	154.04^d3^	133.40 ^e,1^	184.20 ^e,2^	183.27 ^e,2^	157.80 ^f,1^	274.36 ^f,2^	323.97 ^f,3^	345.90 ^g,1^	318.31 ^g,2^	401.05 ^g,3^	8.36
C16:1	6.41 ^a,1^	5.49 ^a,2^	5.82 ^a,3^	8.15 ^b,1^	10.09 ^b,2^	8.55 ^b,3^	8.87 ^c,1^	11.80 ^c,2^	10.54 ^c,3^	10.95 ^d,1^	15.31 ^d,2^	16.32^d3^	16.79 ^e,1^	23.91 ^e,2^	22.35 ^e,3^	29.63 ^f,1^	35.78 ^f,2^	34.99 ^f,3^	43.21 ^g,1^	49.79 ^g,2^	48.96 ^g,3^	1.16
C17	0.65 ^a,1^	0.64 ^a,1^	0.71 ^a,2^	1.06 ^b,1^	1.33 ^b,2^	1.06 ^b,1^	1.14 ^c,1^	1.67 ^b,c,2^	1.32 ^c,3^	1.41 ^d,1^	2.01 ^c,2^	1.44^d3^	2.28 ^e,1^	2.65 ^d,2^	3.43 ^e,3^	2.54 ^f,1^	2.99 ^d,1^	4.56 ^f,2^	4.71 ^g,1^	4.33 ^e,2^	6.61 ^g,3^	0.13
C17:1	0.41 ^a,1^	0.56 ^a,2^	0.49 ^a,3^	0.53 ^b,1^	0.68 ^b,2^	1.15 ^b,3^	0.95 ^c,1^	0.84 ^c,2^	1.43 ^c,3^	1.19 ^d,1^	1.44 ^d,2^	1.74^d3^	1.46 ^e,1^	1.53 ^e,2^	1.82 ^e,3^	1.70 ^f,1^	1.97 ^f,2^	1.91 ^f,3^	2.19 ^g,1^	3.20 ^g,2^	2.40 ^g,3^	0.06
C18	30.98 ^a,1^	25.45 ^a,2^	30.10 ^a,1^	51.51 ^b,1^	74.90 ^b,2^	61.16 ^b,3^	57.53 ^c,1^	94.73 ^c,2^	65.80 ^c,3^	74.64 ^d,1^	103.16 ^d,2^	89.27^d3^	85.67 ^e,1^	150.59 ^e,2^	158.91 ^e,3^	121.79 ^f,1^	158.60 ^f,2^	287.21 ^f,3^	204.19 ^g,1^	202.07 ^g,1^	324.32 ^g,3^	6.40
C18:1n9	70.96 ^a,1^	88.55 ^a,2^	86.48 ^a,2^	123.88 ^b,1^	175.63 ^b,2^	147.32 ^b,3^	148.46 ^c,1^	186.66 ^c,2^	170.44 ^c,3^	213.76 ^d,1^	284.53 ^d,2^	240.09^d3^	296.28 ^e,1^	376.06 ^e,2^	358.92 ^e,3^	446.77 ^f,1^	404.91 ^f,2^	626.05 ^f,3^	654.22 ^g,1^	619.27 ^g,2^	768.83 ^g,3^	16.30
C18:2n6	66.11 ^a,1^	59.90 ^a,2^	63.63 ^a,1^	110.65 ^b,1^	156.17 ^b,2^	121.71 ^b,3^	138.36 ^c,1^	165.10 ^c,2^	163.70 ^c,2^	165.58 ^d,1^	210.54 ^d,2^	184.86^d3^	235.53 ^e,1^	276.81 ^e,2^	249.35 ^e,3^	288.59 ^f,1^	295.00 ^f,2^	475.26 ^f,3^	364.82 ^g,1^	369.26 ^g,2^	515.13^g,3^	9.87
C18:3n6	0.25 ^a,1,2^	0.27 ^a,1^	0.23 ^a,2^	0.38 ^b,1^	0.35 ^b,2^	0.50 ^b,3^	0.39 ^b,1^	0.43 ^c,2^	0.74 ^c,3^	0.46 ^c1^	0.58 ^d,2^	0.85^d3^	0.84 ^d,1^	0.81 ^e,1^	1.27 ^e,3^	1.35 ^e,1^	1.17 ^f,2^	1.52 ^f,3^	1.89 ^g,1^	1.54 ^g,2^	2.01 ^g,3^	0.04
C18:3n3	6.08 ^a,1^	7.71 ^a,2^	7.83 ^a,2^	7.05 ^b,1^	15.51 ^b,2^	11.04 ^b,3^	8.77 ^c,1^	17.44 ^c,2^	12.83 ^c,3^	9.01 ^d,1^	22.32 ^d,2^	12.81^c,3^	16.06 ^e,1^	26.92 ^e,2^	20.07 ^d3^	25.23 ^f,1^	28.82 ^f,2^	26.65 ^e,3^	29.30 ^g,1^	34.54 ^g,2^	28.67 ^f,3^	0.71
C20	0.28 ^a,1^	0.55 ^a,2^	0.48 ^a,3^	0.70 ^b,1^	0.99 ^a,b,2^	1.28 ^b,3^	1.12 ^c,1^	1.10 ^b,c,1^	1.50 ^c,1^	1.07 ^d,1^	1.43 ^b,c,2^	1.63^d3^	1.13 ^c,1^	1.47 ^b,c,2^	2.22 ^e,3^	1.06 ^d,1^	1.77 ^c,d,2^	3.15 ^f,3^	1.75 ^g,1^	2.00 ^d,2^	3.46 ^g,3^	0.06
C20:1n9	1.38 ^a,1^	1.01 ^a,2^	1.35 ^a,1^	2.37 ^b,1^	3.31 ^b,2^	3.39 ^b,3^	2.55 ^c,1^	5.06 ^c,2^	4.60 ^c,3^	4.04 ^d,1^	7.74 ^d,2^	6.92^d3^	6.30 ^e,1^	8.88 ^e,2^	10.21 ^e,3^	8.51 ^f,1^	9.36 ^f,2^	19.09 ^f,3^	12.72 ^g,1^	13.13 ^g,1^	17.65 ^g,2^	0.40
C20:2	1.32 ^a,1^	1.00 ^a,2^	1.38 ^a,3^	2.86 ^b,1^	2.75 ^b,2^	2.58 ^b,3^	3.21 ^c,1^	3.44 ^c,2^	2.93 ^c,3^	4.65 ^d,1^	4.31 ^d,2^	3.48^d3^	6.01 ^e,1^	7.74 ^e,2^	4.15 ^e,3^	7.59 ^f,1^	8.44 ^f,2^	7.49 ^f,1^	11.64 ^g,1^	13.00 ^g,2^	9.24 ^g,3^	0.26
C20:3n6	0.25 ^a,1^	0.34 ^a,2^	0.33 ^a,1^	0.80 ^b,1^	0.65 ^b,2^	0.53 ^b,3^	1.02 ^c,1^	1.06 ^c,1,2^	1.13 ^c,2^	1.67 ^d,1^	2.07 ^d,2^	1.26^d3^	1.87 ^e,1^	2.43 ^e,2^	1.39 ^e,3^	2.39 ^f,1^	4.05 ^f,2^	2.29 ^f,3^	3.50 ^g,1^	4.83 ^g,2^	3.21 ^g,3^	0.10
C20:3n3	0.27 ^a,1^	0.26 ^a,1^	0.22 ^a,3^	0.51 ^b,1^	0.99 ^b,2^	0.88 ^b,3^	0.67 ^c,1^	1.03 ^c,2^	1.08 ^c,3^	1.10 ^d,1^	1.32 ^d,2^	1.63^d3^	2.17 ^e,1^	3.17 ^e,2^	2.74 ^e,3^	2.41 ^f,1^	3.62 ^f,2^	4.82 ^f,3^	2.94 ^g,1^	5.55 ^g,2^	5.53 ^g,2^	0.13
C20:4n6	3.30 ^a,1^	2.41 ^a,2^	3.60 ^a,3^	6.62 ^b,1^	8.24 ^b,2^	6.70 ^b,1^	8.51 ^c,1^	12.85 ^c,2^	11.69 ^c,3^	13.00 ^d,1^	14.73 ^d,2^	16.60^d3^	23.14 ^e,1^	25.93 ^e,2^	27.97 ^e,3^	25.42 ^f,1^	33.82 ^f,2^	53.27 ^f,3^	38.03 ^g,1^	47.86 ^g,2^	51.19 ^g,3^	1.26
C20:5n3	0.17 ^a,1^	0.14 ^a,1^	0.35 ^a,3^	0.37 ^b,1^	0.48 ^b,2^	0.44 ^b,2^	0.60 ^c,1^	0.72 ^c,2^	1.04 ^c,3^	1.03 ^d,1^	1.24 ^d,2^	1.45^d3^	1.33 ^e,1^	1.51 ^e,2^	1.63 ^e,3^	1.27 ^f,1^	1.35 ^f,2^	2.47 ^f,3^	1.92 ^g,1^	2.74 ^g,2^	3.08 ^g,3^	0.07
C22	0.33 ^a,1^	0.48 ^a,2^	0.46 ^a,2^	0.66 ^b,1^	0.82 ^b,2^	0.77 ^b,3^	1.33 ^c,1^	0.93 ^c,2^	1.36 ^c,1^	1.88 ^d,1^	1.87 ^d,1^	1.32^d,2^	1.03 ^e,1^	2.14 ^e,2^	2.58 ^e,3^	1.65 ^f,1^	2.48 ^f,2^	3.71 ^f,3^	2.09 ^g,1^	4.57 ^g,2^	4.17 ^g,3^	0.10
C22:1n9	0.70 ^a,1^	0.59 ^a,2^	0.84 ^a,3^	0.72 ^b,1^	0.89 ^b,2^	1.46 ^b,3^	1.17 ^c,1^	1.12 ^c,1^	1.51 ^c,3^	1.61 ^d,1^	2.35 ^d,2^	1.78^d3^	1.96 ^e,1^	2.80 ^e,2^	3.72 ^e,3^	3.95 ^f,1^	3.33 ^f,2^	4.14 ^f,3^	4.62 ^g,1^	6.29 ^g,2^	5.05 ^g,3^	0.13
C22:2n6	1.55 ^a,1^	2.80 ^a,2^	2.10 ^a,2^	4.16 ^b,1^	4.56 ^b,2^	4.60 ^b,2^	9.22 ^c,1^	5.40 ^c,2^	8.13 ^c,3^	11.90 ^d,1^	9.35 ^d,2^	9.32^d,2^	15.27 ^e,1^	14.25 ^e,2^	14.20 ^e,2^	16.89 ^f,1^	20.73 ^f,2^	21.33 ^f,3^	37.31 ^g,1^	43.34 ^g,2^	34.23 ^g,3^	0.93
C23	0.44 ^a,1^	0.67 ^a,2^	0.56 ^a,3^	0.80 ^b,1^	1.16 ^b,2^	1.05 ^b,3^	1.09 ^c,1^	1.31 ^c,2^	1.65 ^c,3^	2.08 ^d,1^	2.61 ^d,2^	2.94^d3^	3.32 ^e,1^	3.55 ^e,2^	3.61 ^e,3^	3.73 ^f,1^	4.16 ^f,2^	4.72 ^f,3^	6.55 ^g,1^	5.65 ^g,2^	8.52 ^g,3^	0.17
C24	1.44 ^a,1^	1.42 ^a,1^	1.55 ^a,2^	1.98 ^b,1^	1.58 ^b,2^	2.53 ^b,3^	2.40 ^c,1^	3.18 ^c,2^	4.38 ^c,3^	3.68 ^d,1^	3.55 ^d,2^	5.41^d3^	4.59 ^e,1^	6.88 ^e,2^	6.75 ^e,3^	10.95 ^f,1^	15.15 ^f,2^	8.16 ^f,3^	14.95 ^g,1^	26.40 ^g,2^	15.13 ^g,3^	0.52
Total FFA	253.62 ^a,1^	247.11 ^a,1^	263.64 ^a,1^	403.55 ^b,1^	562.72 ^b,2^	469.87 ^b,3^	497.00 ^c,1^	636.29 ^c,2^	601.61 ^c,3^	640.18 ^d,1^	839.84 ^d,2^	767.85^d3^	868.97 ^e,1^	1.142.20 ^e,2^	1.098.57 ^e,3^	1.179.31 ^f,1^	1.333.06 ^f,2^	1.943.04 ^f,3^	1.814.41 ^g,1^	1.805.65 ^g,1^	2.295.46 ^g,2^	46.72
SFA	94.13 ^a,1^	75.60 ^a,2^	88.63 ^a,1,2^	135.20 ^b,1^	181.91 ^b,2^	158.32 ^b,3^	163.42 ^c,1^	222.52 ^c,2^	209.04 ^c,3^	199.28 ^d,1^	260.73 ^d,2^	267.80^d3^	242.70 ^e,1^	367.89 ^e,2^	382.48 ^e,3^	316.20 ^f,1^	478.81^f,2^	660.11 ^f,3^	604.26 ^g,1^	589.16 ^g,2^	798.15 ^g,3^	16.05
UFA	159.49 ^a,1^	171.51 ^a,2^	175.01 ^a,2^	268.35 ^b,1^	380.80 ^b,2^	312.18 ^b,3^	333.58 ^c,1^	413.77 ^c,2^	392.57 ^c,3^	440.90 ^d,1^	579.11 ^d,2^	500.05^d3^	626.27 ^e,1^	774.31 ^e,2^	721.14 ^e,3^	863.11 ^f,1^	854.24^f,2^	1.282.93 ^f,3^	1.210.15 ^g,1^	1.216.49 ^g,1^	1.497.32 ^g,2^	30.90
MUFA	80.2 1^a,1^	96.67 ^a,2^	95.35 ^a,2^	136.18 ^b,1^	191.12 ^b,2^	162.57 ^b,3^	162.85 ^c,1^	206.32 ^c,2^	189.28 ^c,3^	232.46 ^d,1^	312.63 ^d,2^	267.80^d3^	324.03 ^e,1^	414.76 ^e,2^	398.37 ^e,3^	491.97 ^f,1^	457.27^f,2^	687.82 ^f,3^	718.81 ^g,1^	693.84 ^g,2^	845.02 ^g,3^	12.59
PUFA	79.29 ^a,1^	74.83 ^a,2^	79.66 ^a,1^	132.63 ^b,1^	189.81 ^b,2^	149.15 ^b,3^	170.74 ^c,1^	207.46 ^c,2^	203.50 ^c,3^	208.73 ^d,1^	266.73 ^d,2^	237.72 ^d3^	302.23 ^e,1^	359.56 ^e,2^	322.77 ^e,3^	371.57 ^f,1^	396.99 ^f,2^	592.63 ^f,3^	492.08 ^g,1^	522.66 ^g,2^	652.29 ^g,3^	13.18

SFA: sum of saturated fatty acids; UFA: sum of unsaturated fatty acids; MUFA: sum of monounsaturated fatty acids; PUFA: sum of polyunsaturated fatty acids. CNT: Non-inoculated control batches; EQU: Batches inoculated with *L. sakei* + *S. equorum*; SAP: Batches inoculated with *L. sakei* + *S. saprophyticus*. ^a–g^ Means in the same row and sausage group (CNT, EQU or SAP) not followed by a common letter differ significantly (*p* < 0.05) (differences associated to the ripening time). ^1–3^ Means in the same row and ripening time not followed by a common number differ significantly (*p* < 0.05) (differences associated to the use of starter cultures). SEM: standard error of the mean.
